# Histone variant *H3F3A* promotes lung cancer cell migration through intronic regulation

**DOI:** 10.1038/ncomms12914

**Published:** 2016-10-03

**Authors:** Seong-Min Park, Eun-Young Choi, Mingyun Bae, Sunshin Kim, Jong Bae Park, Heon Yoo, Jung Kyoon Choi, Youn-Jae Kim, Seung-Hoon Lee, In-Hoo Kim

**Affiliations:** 1 Specific Organs Cancer Branch, Research Institute, National Cancer Center, Goyang, Gyeonggi 10408, Republic of Korea; 2 Department of Bio and Brain Engineering, KAIST, Daejeon 34141, Republic of Korea; 3 Precision Medicine Branch, Research Institute, National Cancer Center, Goyang, Gyeonggi 10408, Republic of Korea; 4 Department of System Cancer Science, Graduate School of Cancer Science and Policy, National Cancer Center, Goyang, Gyeonggi 10408, Republic of Korea

## Abstract

Although several somatic single nucleotide variations in histone H3.3 have been investigated as cancer drivers, other types of aberration have not been well studied. Here, we demonstrate that overexpression of *H3F3A*, encoding H3.3, is associated with lung cancer progression and promotes lung cancer cell migration by activating metastasis-related genes. H3.3 globally activates gene expression through the occupation of intronic regions in lung cancer cells. Moreover, H3.3 binding regions show characteristics of regulatory DNA elements. We show that H3.3 is deposited at a specific intronic region of *GPR87*, where it modifies the chromatin status and directly activates *GPR87* transcription. The expression levels of *H3F3A* and *GPR87*, either alone or in combination, are robust prognostic markers for early-stage lung cancer, and may indicate potential for the development of treatments involving *GPR87* antagonists. In summary, our results demonstrate that intronic regulation by *H3F3A* may be a target for the development of novel therapeutic strategies.

## Abstract (web summary)

Histone variants act as transcriptional activators and repressors and have been linked to cancer progression. Park and Choi *et al*. show that the histone H3.3 overexpression is associated with early-stage lung cancer, and promotes cancer cell migration by upregulating a G-protein-coupled receptor.

## Graphical abstract

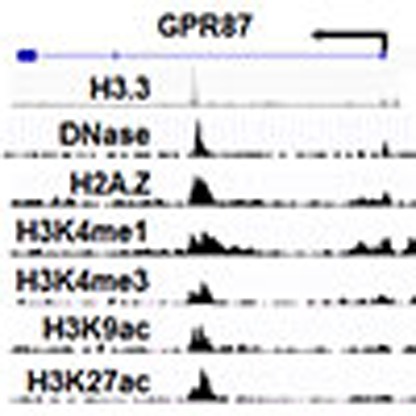

Lung cancer is the most frequent cause of cancer-related death in the world, and patients with non-small cell lung cancer (NSCLC) account for ∼75% of lung cancer cases^1^. The risk of relapse after surgery among NSCLC patients is substantial, with 30–35% of stage I patients relapsing after their initial surgery^2^,^3^. The subgrouping of early-stage lung cancer patients who will face poor prognoses is important for successful treatment including post-operative adjuvant therapy. Conventionally, a histological subtype marker has been used in post-operative adjuvant therapy, but the effectiveness of this approach is controversial^4^,^5^,^6^,^7^. Thus, the identification of novel robust prognostic markers for early-stage lung cancer is imperative.

Histone variants act as transcriptional activators or repressors of cancer-related genes. macroH2A, a non-canonical histone variant, suppresses cancer progression and acts as a direct transcriptional repressor of *CDK8*, a cancer driver gene^8^. H2A.Z, another histone variant, promotes cancer progression via transcriptional regulation in various cancer types^9^,^10^. The histone variant H3.3 is encoded by two genes, *H3F3A* and *H3F3B*. Although only four or five amino acids differ between H3.3 and the canonical histone H3, H3.3 appears to have different functions in gene regulation^11^. H3.3 is highly enriched in the bodies, promoters and enhancers of actively transcribed genes and is associated with active transcription during various biological processes, including processes associated with development and diseases^12^,^13^,^14^,^15^,^16^. Recently, highly recurrent somatic missense mutations of H3.3 genes have been implicated in childhood tumours; in particular, the Lys27Met mutation is a gain-of-function driver mutation that inhibits polycomb repressive complex 2 (PRC2) activity^17^,^18^,^19^,^20^. However, the role of differential expression of the H3.3 genes in tumours, particularly adult tumours, has rarely been studied and remains unknown. H3.3 overexpression has been reported in oesophageal and lung cancer cell lines; however, the function of H3.3 in cancer remains elusive^21^.

Reports have indicated that H3.3 remodels highly ordered chromatin structures in conjunction with various chromatin remodelers, chaperones and other histone marks, including PRC2, HIRA, CTCF and H2A.Z (refs 15, 16, 22, 23, 24, 25). H3.3 plays different roles in gene transcription depending on its binding position in genome. H3.3 actively marks enhancers and determines the transcriptional potential of target genes; moreover, H3.3 on promoter regions results in poised or active transcription^16^. Recently, the importance of intronic DNA regulatory elements (IREs) in transcription has been increasingly revealed^26^,^27^. The transcriptional regulation of IREs is frequently accompanied by histone modification and chromatin remodelling^28^. Certain IREs are associated with cancer progression^28^. Although many researchers have reported the transcriptional regulation activity of H3.3 on promoter and intergenic enhancer regions, transcriptional regulation via the binding of H3.3 to the intronic regions of target genes has not been well studied.

In this study, we demonstrate that the overexpression of H3F3A promotes lung cancer cell migration by activating metastasis-related genes. H3.3, which is encoded by H3F3A, activates gene expression by occupying intronic regions that exhibit characteristics of regulatory DNA elements. H3.3 directly activates GPR87 transcription via its deposition at a specific intronic region of GPR87, where it modifies chromatin status. H3F3A and GPR87 expression levels, either alone or in combination, are associated with lung cancer patients’ prognoses and have potential for the development of treatments involving GPR87 antagonists.

## Results

### *H3F3A* overexpression promotes lung cancer progression

**Figure 1:**
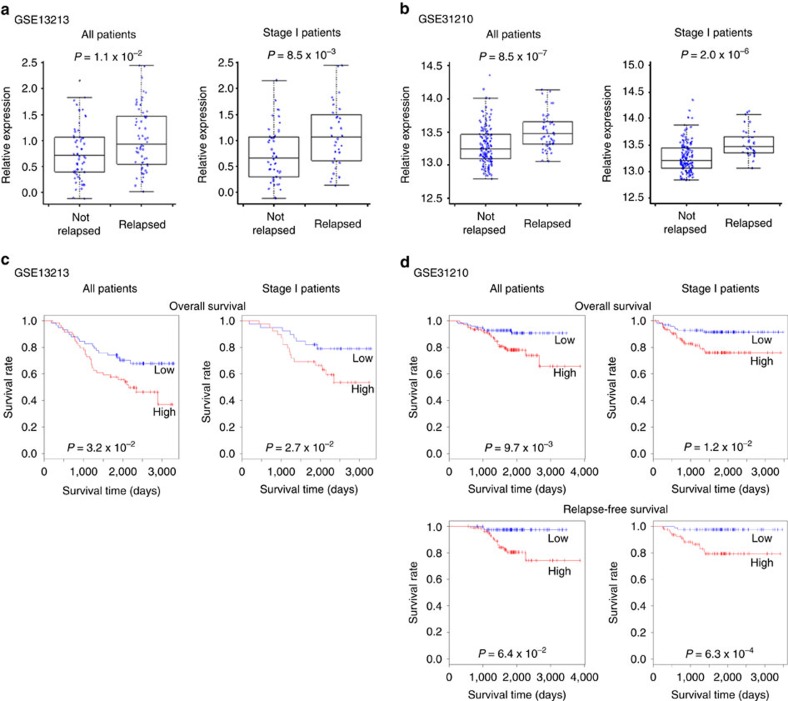
Promotion of lung cancer progression by *H3F3A* overexpression. (**a**) Comparison of *H3F3A* expression in relapsed and non-relapsed lung cancer patients (GSE13213, Tomida *et al*.^29^. All: *n*=117, *P*=1.1 × 10^−2^, stage I: *n*=79, *P*=8.5 × 10^−3^, *t*-test). (**b**) (GSE31210, Okayama *et al*.^30^. All: *n*=204, *P*=8.5 × 10^−7^, stage I: *n*=162, *P*=2.0 × 10^−6^, *t*-test). (**c**) Prognosis of two groups of lung cancer patients classified by *H3F3A* expression (GSE13213, Tomida *et al*.^29^. All: *n*=117, *P*=3.2 × 10^−2^, stage I: *n*=79, *P*=2.7 × 10^−2^, log-rank test). (**d**) (GSE31210, Okayama *et al*.^30^. All: *n*=204, *P*=9.7 × 10^−3^ for overall survival, *P*=6.4 × 10^−2^ for relapse-free survival; stage I: *n*=162, *P*=1.2 × 10^−2^ for overall survival, *P*=6.3 × 10^−4^ for relapse-free survival, log-rank test). Red: high expression group, blue: low expression group.

Previous studies addressing the role of H3.3 genes in cancer have primarily focused on recurrent mutations. However, a variety of point mutations and copy number variations (CNV) in H3.3 genes are found at a low frequency in lung cancer samples, and many well-characterized mutations such as Lys27Met are not recurrent in lung cancer according to publicly available lung cancer data sets from the Catalogue of Somatic Mutations in Cancer database (http://cancer.sanger.ac.uk/cancergenome/projects/cosmic/, COSMIC; H3F3A: 3/1554 mutations and 15/827 CNV; H3F3B: 4/1553 mutations and 9/827 CNV). Therefore, we examined the association between H3.3 expression and lung cancer progression and evaluated the possibility of using H3.3 genes as prognostic markers for lung cancer. We collected and analysed public microarray lung cancer data sets from the NCBI Gene Expression Omnibus (GEO) database containing clinical information such as cancer stage, recurrence status and survival data. Two data sets from lung adenocarcinomas, GSE13213 and GSE31210, were used in this study because they were already published, and clinical information from a sufficient number of samples was available^29^,^30^. We performed the analyses using data from all of the patients in each data set (117 patients in GSE13213 and 204 patients in GSE31210) or only data from stage I patients (79 patients in GSE13213 and 162 patients in GSE31210). Comparing H3.3 gene (*H3F3A* and *H3F3B*) expression in non-relapsed and relapsed patients revealed that *H3F3A* expression was significantly higher in the relapsed patient group in both data sets (all: *P*=1.1 × 10^−2^ for GSE13213 and *P*=8.5 × 10^−7^ for GSE31210; stage I: *P*=8.5 × 10^−3^ for GSE13213 and *P*=2.0 × 10^−6^ for GSE31210, *t*-test), whereas *H3F3B* expression was only significantly higher among relapsed patients in the GSE31210 data set (Fig. 1a,b, Supplementary Fig. 1a). Using the survival data, we investigated the association between the survival rate for lung adenocarcinoma patients after surgery and H3.3 gene expression (Fig. 1c,d, Supplementary Fig. 1b). Survival was significantly lower in the group with higher *H3F3A* expression than it was in the lower expression group in both data sets (all: *P*=3.2 × 10^−2^ for GSE13213, *P*=9.7 × 10^−3^ for GSE31210 overall survival, *P*=6.4 × 10^−2^ for GSE31210 relapse-free survival; stage I: *P*=2.7 × 10^−2^ for GSE13213, *P*=1.2 × 10^−2^ for GSE31210 overall survival, *P*=6.3 × 10^−4^ for GSE31210 relapse-free survival, log-rank test), whereas the higher *H3F3B* expression group exhibited significantly lower survival only in the GSE31210 data set. The expression of canonical histone H3 genes was not significantly associated with relapse or survival (Supplementary Figs 2 and 3). Based on these results, we suggest that *H3F3A* overexpression in lung cancer promotes cancer progression, and we subsequently focused on *H3F3A* and stage I lung cancer.

To assess the value of *H3F3A* expression as a prognostic marker for stage I lung adenocarcinoma, we performed a multivariate Cox proportional hazard analysis using the two public data sets (Table 1). The level of *H3F3A* expression better predicted poor survival (low survival rate) in both data sets than other markers, including histological grouping into stage IA and stage IB. Thus, we suggest that *H3F3A* overexpression is a good prognostic marker for early-stage lung cancer.

### *H3F3A* overexpression promotes cancer cell invasion

**Figure 2:**
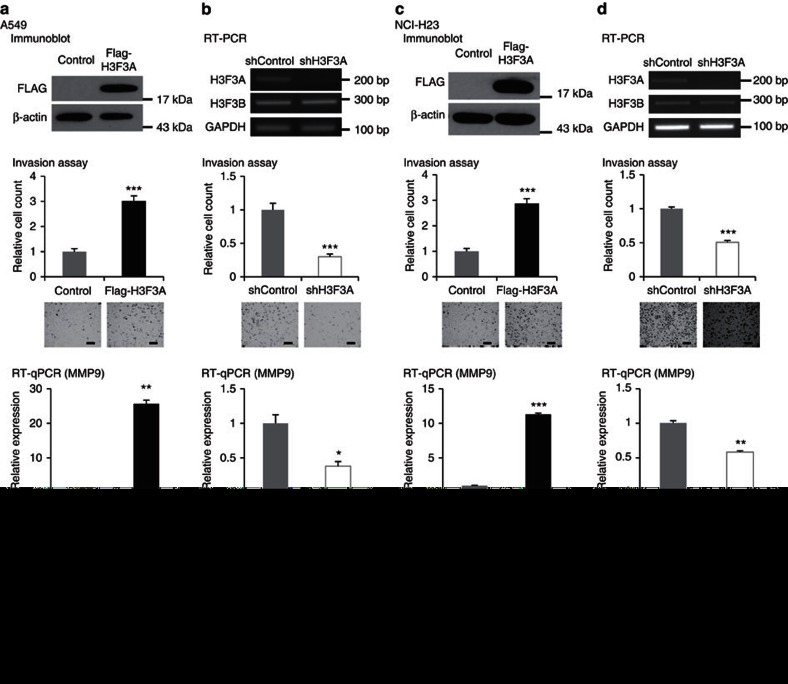
Promotion of lung cancer cell invasion by *H3F3A* overexpression. (**a**) Results of invasion assay, *MMP9* RT-qPCR and proliferation assay after ectopic *H3F3A* overexpression in A549 cells. (**b**) After *H3F3A* knockdown in A549 cells. (**c**) After ectopic *H3F3A* overexpression in NCI-H23 cells. (**d**) After *H3F3A* knockdown in NCI-H23 cells. Data are representative of three independent experiments. The error bars represent the s.e.m. *P<0.05, **P<0.01, ***P<0.001, *t*-test. Scale bar, 200 μm.

Additional clinical information about relapse sites was available in the GSE13213 data set, which revealed that all but six relapsed patients exhibited distant metastases. As *H3F3A* overexpression was associated with relapse and poor prognosis (low survival rate) in lung cancer patients, we ectopically overexpressed *H3F3A* in human lung cancer cell lines and examined several cancer-related phenotypes. To evaluate metastasis-related phenotypes, we performed an invasion assay using *H3F3A*-overexpressing A549 and NCI-H23 cells (Fig. 2a,c). *H3F3A* overexpression significantly increased lung cancer cell invasion (*P*=2.0 × 10^−5^ for A549 and *P*=7.7 × 10^−6^ for NCI-H23, *t*-test). We also performed an invasion assay using *H3F3A*-knockdown A549 and NCI-H23 cells (Fig. 2b,d). As expected, *H3F3A* knockdown significantly decreased invasion (*P*=1.0 × 10^−5^ for A549 and *P*=1.7 × 10^−8^ for NCI-H23, *t*-test). Furthermore, we performed invasion assays using canonical histone H3-overexpressing A549 cells (Supplementary Fig. 4). Overexpression of the H3.1 and H3.2 genes did not have significant effects on the invasion of A549 cells. Next, we investigated changes in the expression of *MMP9*, a well-known invasion-related effector gene, following *H3F3A* overexpression or knockdown (Fig. 2). As expected, *MMP9* expression was increased by *H3F3A* overexpression (overexpression: *P*=1.7 × 10^−3^ for A549 and *P*=9.7 × 10^−5^ for NCI-H23, *t*-test; knockdown: *P*=3.8 × 10^−2^ for A549 and *P*=1.3 × 10^−3^ for NCI-H23, *t*-test). We next examined growth-related phenotypes by performing cell proliferation assays, although we did not observe any significant changes in proliferation following *H3F3A* overexpression or knockdown (Fig. 2, bottom). Based on these results, we concluded that *H3F3A* overexpression in lung cancer cells promotes cancer cell invasion, which is important for cancer metastasis and progression.

### Transcriptional regulation by *H3F3A* in lung cancer

**Figure 3:**
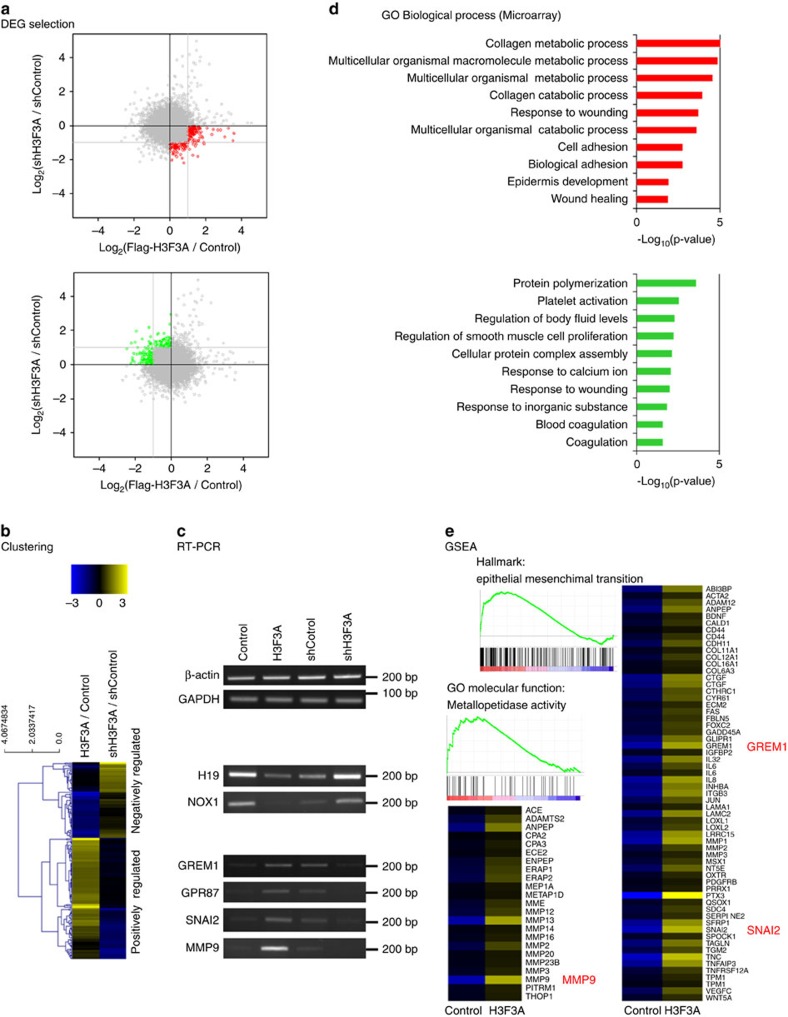
Microarray-based global analysis for transcriptional regulation via *H3F3A.* (**a**) Selection of DEGs using the expression microarray analysis in *H3F3A*-altered A549 cells. (**b**) Expression pattern of the DEGs. (**c**) Validation of DEGs using RT-PCR. (**d**) GO analysis result of the DEGs (GO_BP). (**e**) Gene set enrichment analysis result of the positively correlated DEGs.

*H3F3A* encodes the histone variant H3.3, the general function of which is to regulate transcription. Thus, we hypothesized that H3.3 functions in lung cancer by transcriptionally regulating downstream target genes. To identify *H3F3A* target genes, we performed microarray-based global gene expression analysis of *H3F3A*-overexpressing and *H3F3A* knockdown A549 cells. Genes that exhibited >2-fold changes in expression following either overexpression or knockdown, with opposite responses in the overexpression and knockdown treatments, were identified as differentially expressed genes (DEGs) (Fig. 3a,b). A total of 112 positively regulated DEGs (Supplementary Data 1) and 70 negatively regulated DEGs were selected (Supplementary Data 2). The expression patterns of some candidate genes were validated by quantitative reverse transcription PCR (RT-qPCR; Supplementary Fig. 5) and semi-quantitative RT-PCR (RT-PCR; Fig. 3c). We performed Gene Ontology (GO) analysis of the selected DEGs (Fig. 3d and Supplementary Data 3 and 4), and the top-ranked Gene Ontology term among the genes that were positively regulated by *H3F3A* expression was ‘collagen metabolic process,’ which included several well-known metastasis-related effector genes, such as metallomatrix proteases. We did not observe any metastasis-related terms for the negatively regulated genes, leading us to focus on the positively regulated genes in the subsequent analysis. Following further analysis using the Gene Set Enrichment Analysis programme, we also found that genes that were positively regulated by *H3F3A* were significantly associated with the ‘Epithelial Mesenchymal Transition’ and ‘Metallopetidase Activity’ gene sets, which are closely involved in cancer metastasis (Fig. 3e). Therefore, we concluded that genes that are positively regulated by *H3F3A* are closely associated with metastasis-related processes.

**Figure 4:**
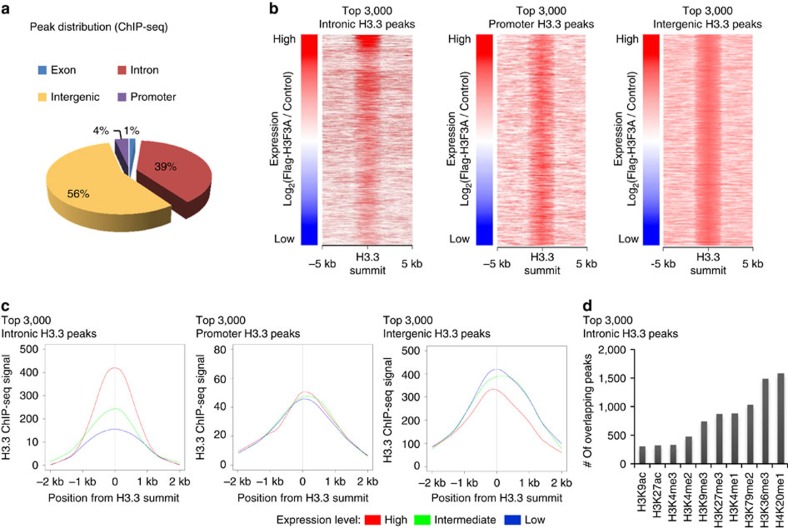
Chip-seq-based global analysis for transcriptional regulation via *H3F3A*. (**a**) Genomic distribution of H3.3 ChIP-seq peaks in *H3F3A*-overexpressed A549 cells. (**b**) Correlation between H3.3 occupancy and target gene expression at intron, promoter and intergenic region. (**c**) H3.3 binding profile of intronic/promoter/intergenic peaks of three gene groups classified by the expression level (red: high expression group, yellow-green: intermediate expression group, blue: low expression group). (**d**) Association of H3.3 peaks with various histone marks.

To identify genes that are occupied by H3.3 following *H3F3A* overexpression, we performed chromatin immunoprecipitation (ChIP)-seq using *H3F3A*-overexpressing A549 lung cancer cells. In total, 871 H3.3 peaks were detected following overexpression using a high stringency cutoff, and we then annotated these peaks by genomic position (Fig. 4a). The majority of the peaks localized to intergenic and intronic regions (intergenic: ∼56%, intronic: ∼39%). To examine the correlation between H3.3 occupancy and target gene expression, we identified the top 3,000 peaks that were located within introns, promoters and intergenic regions using a low-stringency cutoff. The target genes of intergenic peaks could not be identified without additional data. Thus, we defined the nearest genes within 10 kb from the peaks as the target genes, assuming that the peaks corresponded to *cis*-regulatory elements. We examined the H3.3 ChIP-seq read distribution near each H3.3 summit, and after calculating a normalized read count around each of the 3,000 H3.3 summits located within introns, promoters and intergenic regions (from −5 kb to +5 kb), we sorted the peaks based on target gene expression level (Fig. 4b). Additionally, after separating the peaks into three groups based on the expression of the target genes (high, intermediate and low expression groups), we calculated average normalized read counts for these groups near the H3.3 summits (from −2 kb to +2 kb; Fig. 4c). In both analyses, H3.3 intronic peaks were positively correlated with target gene expression, whereas promoter and intergenic peaks showed no such correlation. Thus, it appears that H3.3 occupancy of introns activates the expression of target genes.

To perform a further analysis, we downloaded histone ChIP-seq data from the Encyclopedia of DNA Elements (ENCODE) database (https://genome.ucsc.edu/ENCODE/). Because H3.3 binding at introns was correlated with target gene expression, we examined with which histone marks H3.3 was associated (Fig. 4d). In A549 lung cancer cells, the top 3,000 intronic H3.3 peaks most frequently overlapped with peaks of mono-methylation of histone H4 lysine 20 (H4K20me1), tri-methylation of histone H3 lysine 36 (H3K36me3) and di-methylation of histone H3 lysine 79 (H3K79me2), as these marks are gene body histone marks. Aside from the gene body marks, intronic H3.3 most frequently bound to the enhancer-specific histone mark mono-methylated histone H3 lysine 4 (H3K4me1), suggesting that intronic H3.3 tends to bind enhancers.

### Transcriptional regulation of *GPR87* by *H3F3A*

**Figure 5:**
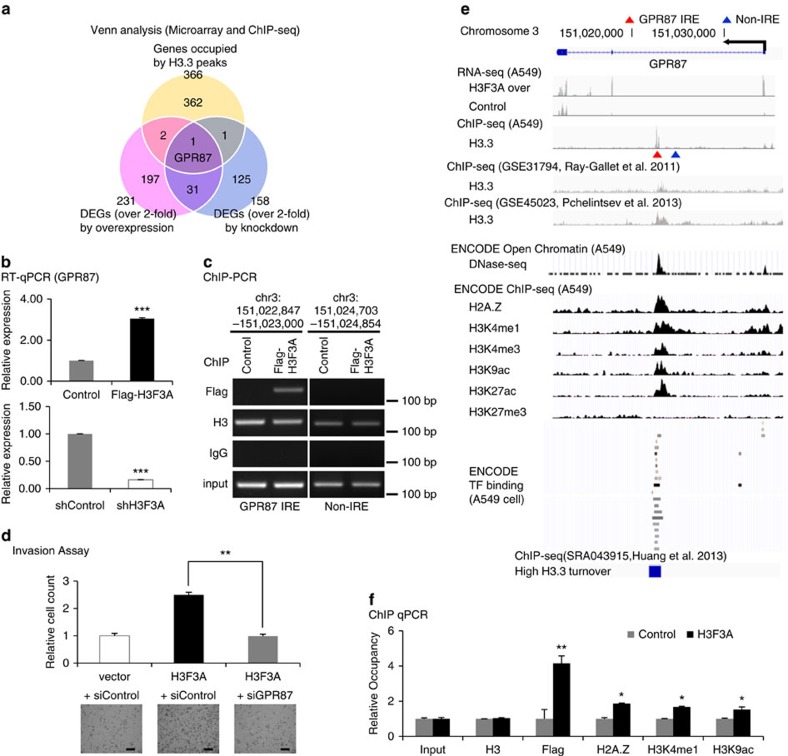
Selection and validation of an H3.3 target gene. (**a**) Selection of putative direct target genes of *H3F3A* with microarray and ChIP-seq data. (**b**) RT-qPCR validation of the changes in the expression of *GPR87* induced by *H3F3A* overexpression and knockdown. (**c**) Validation of H3.3 binding to the IRE of *GPR87* using a ChIP-PCR assay (*GPR87* IRE: chr3:151,022,847-151,023,000; Non-IRE: chr3:151,024,703-151,024,854). (**d**) Results of invasion assays after ectopic *H3F3A* overexpression and *GPR87* knockdown. (**e**) Integrated analysis near *GPR87* gene. (**f**) Chromatin status modification at *GPR87* IRE due to ectopic *H3F3A* overexpression. Data are representative of three independent experiments. The error bars represent the s.e.m. *P<0.05, **P<0.01, ***P<0.001, *t*-test. Scale bar, 200 μm.

To select direct transcriptional targets of *H3F3A*, we performed a Venn analysis using the ChIP-seq and microarray data. Considering that intronic H3.3 frequently bound to enhancer-specific histone mark H3K4me1, as shown in Fig. 4d, we selected 787 genes occupied by both H3.3 and H3K4me1 peaks from the 2,392 genes that are occupied by the top 3,000 intronic H3.3 peaks. Performing Venn analysis with the 787 genes from ChIP-seq and the DEGs using low-stringency cutoffs (top 3,000 probes) from the microarray, we found 29 putative target genes exhibiting H3.3-occupied intronic regulation (Supplementary Fig. 6). Using high stringency cutoffs, we performed another Venn analysis. Performing Venn analysis with the 366 genes occupied by the high stringency H3.3 peaks and the DEGs showing >2-fold changes in expression following H3F3A overexpression or knockdown, we selected one putative target gene, G-protein-coupled receptor 87 (*GPR87*) (Fig. 5a). *GPR87* was also included in the 29 low-stringency putative target genes. The differential expression of *GPR87* by *H3F3A* overexpression and knockdown was validated using RT-qPCR (Fig. 5b). Moreover, we confirmed specific H3.3 binding to the *GPR87* intron using a ChIP-PCR assay (Fig. 5c). As *GPR87* is known to be associated with cancer progression in NSCLC (ref. 31), we focused on *GPR87* in the subsequent analysis.

As we expected that *H3F3A* would promote lung cancer cell invasion through *GPR87*, we examined whether the increased invasion ability stimulated by *H3F3A* overexpression could be mitigated by *GPR87* knockdown (Fig. 5d). *GPR87* knockdown strongly suppressed the increased invasion ability induced by *H3F3A* overexpression (*P*=2.0 × 10^−3^, *t*-test). In addition, double *H3F3A*/*GPR87* knockdown did not further potentiate the effect on the invasion of single knockdowns (Supplementary Fig. 7). We concluded that increased *GPR87* expression caused by *H3F3A* overexpression promotes the invasion ability of lung cancer cells.

Occupancy of H3.3 at the *GPR87* intronic region has been observed in various public data sets, such as NCBI GEO GSE31794 and GSE45023 data sets for HeLa cervical cancer cells^23^,^32^ (Fig. 5e). H3.3 ChIP-seq data sets for lung cancer cells were not available, prompting us to use H3.3 ChIP-seq data sets from HeLa cervical cancer cells. The integrated analysis suggested that the H3.3-occupied *GPR87* intronic region is an intronic regulatory DNA element (IRE), as it is located in an open chromatin region that is bound to more transcription factors than the *GPR87* promoter region, and is also occupied by active histone marks such as H2A histone family member Z (H2A.Z), mono- and tri-methylation of histone H3 lysine 4 (H3K4me1 and H3K4me3) and acetylation of histone H3 lysine 9 and lysine 27 (H3K9ac and H3K27ac) (Fig. 5e). Tri-methylation of histone H3 lysine 27 (H3K27me3), a repressive histone mark, was not observed in this region. To examine changes in chromatin status at the *GPR87* IRE, we performed a ChIP-qPCR assay to analyse changes in several histone marks (Fig. 5f). *H3F3A* overexpression increased the occupancy of three types of histone marks: H2A.Z (an active histone variant), H3K4me1 (an enhancer-specific histone mark) and H3K9ac (a histone mark of active promoters and enhancers)^33^. These results indicate that *H3F3A* overexpression modifies the chromatin status near the *GPR87* IRE, demonstrating that this is a true H3.3-occupied IRE.

To perform a further integrated analysis, we downloaded another public H3.3 ChIP-seq data set from the NCBI sequence read archive (SRA) database: SRA043915 (ref. 34). The study of SRA043915 allowed us to separate high H3.3 nucleosome turnover regions from the rest of the genome. Interestingly, GPR87 IRE overlapped with a high H3.3 nucleosome turnover region (Fig. 5e), which was reported to be associated with active histone modification marks at promoters and enhancers^35^, consistent with our result. Then, we performed another global analysis using the SRA043915 data, our own H3.3 ChIP-seq data for A549 cells and the ENCODE ChIP-seq data for A549 cells. After calculating normalized read counts for histone marks near each of the 871 high-stringency H3.3 summits (from −1 kb to +1 kb), we compared the occupancy of histone marks on H3.3 peaks that overlapped with high turnover regions with that for low turnover regions (Supplementary Fig. 8). The H3.3 peaks that overlapped with high turnover regions showed higher occupancy of active histone marks, such as H2A.Z, H3K4me1, H3K4me3, H3K9ac and H3K27ac, and lower occupancy for repressive histone marks, such as tri-methylation of histone H3 lysine 9 (H3K9me3) and H3K27me3, compared with the H3.3 peaks that overlapped with low turnover regions. The high turnover H3.3 peaks show characteristics of active regulatory DNA elements. These results are also consistent with the previous report^35^.

**Figure 6:**
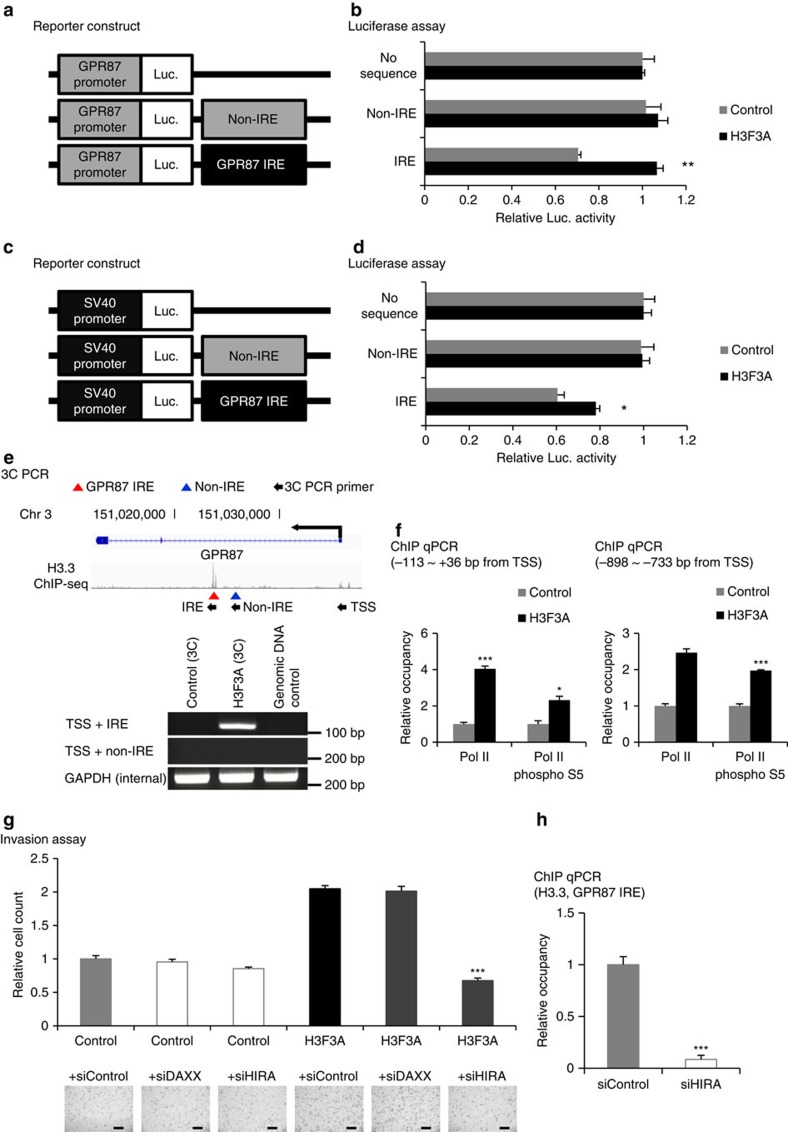
Transcriptional regulation of *GPR87* by *H3F3A.* (**a**) The element-directed reporter construct (*GPR87* promoter). (**b**) The results of luciferase assays using the reporter (*GPR87* promoter). (**c**) The element-directed reporter construct (*SV40* promoter). (**d**) The results of luciferase assays using the reporter (*SV40* promoter). (**e**) Validation of chromatin interaction changes between the *GPR87* IRE and TSS due to *H3F3A* overexpression. (**f**) Validation of transcriptional machinery activation at the TSS by *H3F3A* overexpression. (**g**) Invasion assay with *H3F3A* overexpression after *DAXX* or *HIRA* knockdown. (**h**) ChIP-qPCR assay with *H3F3A* overexpression after *HIRA* knockdown (Flag ChIP at *GPR87* IRE). Data are representative of three independent experiments. The error bars represent the s.e.m. *P<0.05, **P<0.01, ***P<0.001, *t*-test. Scale bar, 200 μm.

The previous results indicated that the *GPR87* IRE could be an intronic hotspot for transcriptional regulation. To examine the functional role of the *GPR87* IRE, we constructed reporters containing the *GPR87* IRE (154 bp), a non-IRE sequence (152 bp, located at 1,703 bp upstream from the *GPR87* IRE) and no sequence (Fig. 6a,c). The *GPR87* and *SV40* promoters were inserted into the constructs to examine promoter dependence. Luciferase assays using these reporters revealed that the *GPR87* IRE suppressed reporter gene expression, whereas the non-IRE sequence had no effect (Fig. 6b,d). Interestingly, *H3F3A* overexpression at least partially rescued this suppression of gene expression (Fig. 6b,d). The effects of both *GPR87* IRE and *H3F3A* overexpression were independent of promoter type. Therefore, we concluded that *H3F3A* overexpression increases *GPR87* expression by suppressing the inhibitory activity of the *GPR87* IRE.

To examine chromatin interactions between the *GPR87* IRE and transcription start site (TSS), we performed a chromatin conformation capture (3C) PCR assay with primers specific to the *GPR87* IRE, non-IRE and TSS (Fig. 6e). *H3F3A* overexpression increased chromatin interactions between the *GPR87* IRE and TSS, suggesting that H3.3 binding to the *GPR87* IRE generates a physical interaction between the *GPR87* IRE and TSS. Next, we evaluated whether changes at the *GPR87* IRE caused by H3F3A overexpression influence the transcriptional machinery at the *GPR87* TSS. By performing a ChIP-qPCR assay near the TSS, we found that *H3F3A* overexpression increased the occupancy of RNA polymerase II (Pol II) and phosphorylated RNA polymerase II (Pol II phospho S5) at the proximal (−113 to +36 bp around the TSS) and distal (−898 to −733 bp upstream of the TSS) *GPR87* promoters (Fig. 6f). These results demonstrate that H3.3 binding to the *GPR87* IRE directly regulates the transcriptional machinery at *GPR87*.

*HIRA* and *DAXX/ATRX* are known as the histone chaperone complex, which recruits assembly factors for H3.3 deposition^15^,^24^. To determine which H3.3 chaperone is involved in regulating invasion ability, we performed invasion assays with *H3F3A*-overexpressing cells after *DAXX* and *HIRA* short interfering RNA (siRNA) treatment (Fig. 6g). Knockdown of *DAXX* did not influence invasion ability, but *HIRA* knockdown significantly decreased the enhanced invasion caused by H3.3 overexpression. To examine whether *HIRA* is involved in promoting H3.3 loading at the *GPR87* IRE, we performed a ChIP-qPCR assay with *H3F3A* overexpression after *HIRA* knockdown (Fig. 6h). Knockdown of *HIRA* significantly decreased H3.3 binding at the *GPR87* IRE. These results imply that *HIRA* acts as a chaperone for H3.3 loading at the *GPR87* IRE. The expression levels of *DAXX* and *HIRA* were not significantly associated with the survival of lung cancer patients (Supplementary Fig. 9).

### Lung cancer promotion by the combination of *H3F3A* and *GPR87*

**Figure 7:**
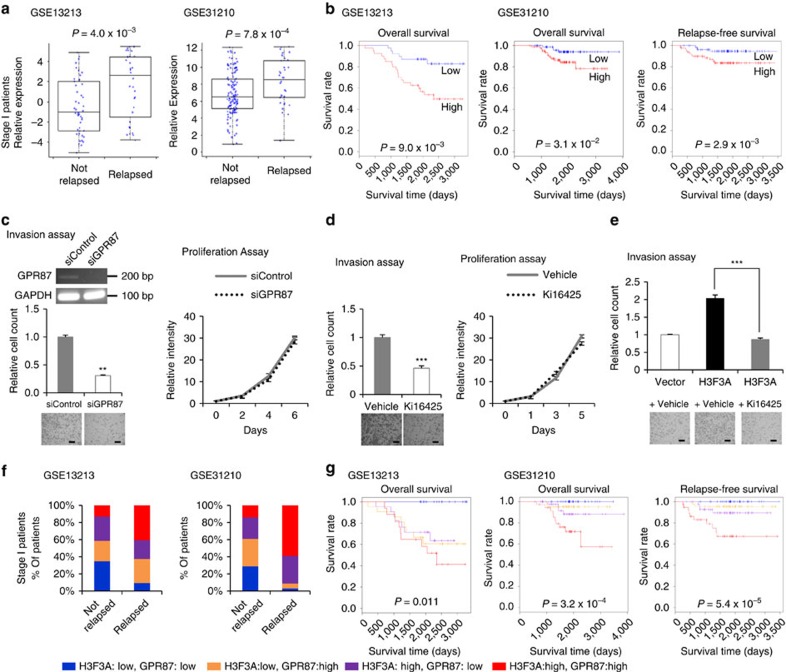
Promotion of lung cancer by *H3F3A* and *GPR87* combination. (**a**) Comparison of *GPR87* expression in relapsed and non-relapsed lung cancer patients. (*P*=4.0 × 10^−3^ for GSE13213 (*n*=79) and *P*=7.8 × 10^−4^ for GSE31210 (*n*=162), *t*-test). (**b**) Prognosis of two groups of lung cancer patients classified by *GPR87* expression. (*P*=9.0 × 10^−3^ for GSE13213 (*n*=79), 3.1 × 10^−2^ for overall survival of GSE31210 (*n*=162) and *P*=2.9 × 10^−3^ for relapse-free survival of GSE31210 (*n*=162), log-rank test; red: high expression group, blue: low expression group). (**c**) Results of invasion and proliferation assays after *GPR87* knockdown. (**d**) Results of invasion and proliferation assays after LPA receptor antagonist (Ki16425) treatment. (**e**) Results of invasion assays after ectopic *H3F3A* overexpression and LPA receptor antagonist (Ki16425) treatment. (**f**) Comparison of the portion of stage I lung adenocarcinoma patient groups stratified by *H3F3A* and *GPR87* expression between non-relapsed and relapsed patients. (**g**) Prognosis of stage I lung adenocarcinoma patient groups stratified by *H3F3A* and *GPR87* expression. (Blue: low *H3F3A*, low *GPR87*; orange: low *H3F3A*, high *GPR87*; purple: high *H3F3A*, low *GPR87*; red: high *H3F3A*, high *GPR87*). Data are representative of three independent experiments. The error bars represent the s.e.m. *P<0.05, **P<0.01, ***P<0.001, *t*-test. Scale bar, 200 μm.

Based on previous results, the transcriptional regulation of *GPR87* by *H3F3A* appears to be essential for the invasion ability of lung cancer cells. Thus, we attempted to characterize the clinical relevance of *GPR87* transcriptional regulation by *H3F3A* overexpression. An analysis of the data sets shown in Fig. 1 revealed that *GPR87* expression is significantly associated with both relapse and prognosis in stage I lung adenocarcinoma patients (Fig. 7a,b; relapse: *P*=4.0 × 10^−3^ for GSE13213 and *P*=7.8 × 10^−4^ for GSE31210, *t*-test; survival: *P*=9.0 × 10^−3^ for GSE13213, 3.1 × 10^−2^ for overall survival of GSE31210 and *P*=2.9 × 10^−3^ for relapse-free survival of GSE31210, log-rank test). Therefore, we suggest that *GPR87* expression changes driven by *H3F3A* overexpression promote lung cancer progression.

*GPR87* is a G-protein-coupled receptor essential for the development and maintenance of tumours and is a promising novel target for cancer treatment^36^. To determine whether *GPR87* influences cancer-related phenotypes, we performed invasion and proliferation assays using *GPR87*-knockdown cells. Similar to *H3F3A*, *GPR87* knockdown significantly decreased lung cancer cell invasion (*P*=1.5 × 10^−3^, *t*-test) and *MMP9* expression (*P*=9.5 × 10^−3^, *t*-test), but did not affect proliferation ability (Fig. 7c and Supplementary Fig. 10). As lysophosphatidic acid (LPA) is a well-known *GPR87* ligand, Ki16425, an LPA receptor antagonist, has been used for *GPR87* inhibition in other studies^36^,^37^,^38^. Thus, we examined whether treatment with the LPA receptor antagonist influenced the invasion and proliferation ability of lung cancer cells (Fig. 7d). Similar to *GPR87* knockdown, treatment with Ki16425 significantly decreased the invasion ability of A549 lung cancer cells, whereas it did not significantly affect proliferation ability. We also examined whether the increased invasion ability stimulated by *H3F3A* overexpression could be blocked by the LPA receptor antagonist Ki16425 (Fig. 7e). Interestingly, treatment with Ki16425 strongly suppressed the increased invasion ability induced by *H3F3A* overexpression (*P*=5.2 × 10^−6^, *t*-test). In addition, Ki16425 treatment after GPR87 knockdown did not cause dramatic further inhibition of invasion ability, suggesting that the effect of Ki16425 is relatively specific (Supplementary Fig. 11). Therefore, targeting *GPR87* in *H3F3A-*overexpressing lung cancer patients might be a viable strategy for post-operative adjuvant therapy.

Analysing the same data sets presented in Fig. 1, we examined the effect of the combination of *H3F3A*/*GPR87* on relapse and patient prognosis. We divided the patients into four groups according to their *H3F3A* and *GPR87* expression levels and then compared the portion of the four group patients between non-relapsed and relapsed patients (Fig. 7f). The relapsed patients contained a high proportion of patients in the high *H3F3A* and high *GPR87* expression group, and a low proportion in the low *H3F3A* and low *GPR87* expression group in the both data sets. We also performed a survival analysis (Fig. 7g and Supplementary Table 1). Surprisingly, all of the patients in the low *H3F3A* and low *GPR87* expression group were still alive. The patients in the high *H3F3A* and high *GPR87* expression group exhibited the worst prognoses. These results imply that the increase in *GPR87* expression induced by *H3F3A* overexpression plays a critical role in lung cancer progression. Thus, we suggest that the combination of *H3F3A*/*GPR87* expression is a novel prognosis marker for early-stage lung cancer.

## Discussion

Histone variants can act as cancer drivers through the transcriptional activation or repression of cancer-related genes. For example, macroH2A suppresses melanoma progression by repressing the transcription of the cancer driver gene *CDK8* (ref. 8). In this study, *H3F3A* affects cancer progression by directly regulating the cancer driver gene *GPR87*, although in this case, it promotes cancer progression by acting as a transcriptional activator. Similar to *H3F3A*, the histone variant H2A.Z promotes cancer through transcriptional activation in various cancer types. The incorporation of H2A.Z into the promoter regions of oestrogen receptor (ERalpha) target genes (for example, *TFF1*) activates transcription in the presence of oestrogen in breast cancer cells^9^, and one isoform of H2A.Z mediates proliferation and drug sensitivity in melanoma by interacting with *BRD2* and *E2F* target genes^39^. Regulatory regions containing both H3.3 and H2A.Z are known to be nucleosome-free regions that play distinct roles in transcriptional modulation^16^,^22^. According to our results, H3.3 is deposited at the *GPR87* IRE, where it increases the occupancy of H2A.Z, modifies chromatin status and interactions, and directly activates *GPR87* transcription. Therefore, this finding is a novel example of a histone variant acting as a transcriptional activator of cancer-promoting genes.

The well-known Lys27Met mutation in H3.3 acts as gain-of-function driver by inhibiting PRC2 activity in brain tumours^17^,^18^,^19^,^20^. Nevertheless, the Lys27Met mutation does not appear to be recurrent in lung cancer based on public cancer-related databases. Although the Lys27Met mutation indirectly upregulates the expression of target genes by inhibiting repressive H3K27me3 modifications, H3.3 overexpression in lung cancer directly upregulates the expression of *GPR87* by promoting the active histone modifications H3K4me1 and H3K9ac, which are known to be important enhancer-associated chromatin modifications^33^. These results demonstrate that H3.3 overexpression increased the *GPR87* IRE enhancer activity, which is a novel example of a direct mechanism enhancing the transcription of cancer-promoting genes.

In general, the function of H3.3 at promoter and intergenic enhancer regions has been relatively well characterized. At promoter regions, H3.3 primes and/or activates transcription by counteracting chromatin compaction, whereas at enhancers, it affects the transcriptional potential of target genes^16^. Although it is known that IREs have a large impact on transcriptional regulation by affecting histone modifications and chromatin remodelling, transcriptional regulation through H3.3 binding to the intronic regions of target genes has not been well studied^26^,^27^,^28^. Based on our integrated analyses, H3.3 deposition at intronic regions globally activates target gene expression. Moreover, globally, H3.3 binding regions showed characteristics of regulatory DNA elements and tended to contain active transcription histone marks. These results suggest that H3.3 is an important global regulator of transcription that can act through IREs.

*GPR87* encodes a G-protein-coupled receptor and is a viable target molecule for cancer treatment and prevention^36^. *GPR87* has been reported to promote growth, p53-dependent cell survival and the metastasis of human tumour cells in a variety of cancers^31^,^40^,^41^,^42^. However, upstream regulators of *GPR87* have not been well characterized. We demonstrate that *GPR87* expression is upregulated by *H3F3A* overexpression and that H3.3 (expressed from *H3F3A*) is deposited at a specific intronic region of *GPR87* in lung cancer. Moreover, by performing element-directed reporter and 3C-PCR assays, we describe in detail how H3.3 modifies the chromatin status of the *GPR87* IRE to regulate the transcriptional activity of this gene. Our results reveal a novel upstream *GPR87* regulator and elucidate an important transcriptional regulation mechanism in the context of lung cancer.

Two distinct genes, *H3F3A* and *H3F3B*, both encode the identical protein H3.3, although they appear to be differentially regulated in cancer. For example, *H3F3A* and *H3F3B* show distinct recurrent mutations in cancer samples^43^, and they also possess different 5′ and 3′ UTR, exon–intron and promoter structures. Furthermore, *H3F3A* and *H3F3B* appear to be regulated by different transcriptional and post-transcriptional factors, such as microRNAs. We found that *H3F3B* was associated with lung cancer progression in one data set but not in the other. We believe this result might have been due to differences between the two microarray platforms used for the GSE13213 and GSE31210 data sets. In particular, GSE13213 used a two-channel microarray platform (normal-tumour difference), whereas GSE31210 used a one-channel microarray platform (expression in tumours). Nevertheless, *H3F3A* showed a strong association with lung cancer progression in both data sets, indicating that *H3F3A* is a robust prognostic marker.

Although the phenotypes were validated only for *in vitro* models and not for *in vivo* models, *H3F3A* and *GPR87* both specifically influenced metastasis-related phenotypes but not growth-related phenotypes, suggesting that they mainly influence cancer progression and not tumorigenesis. Either gene alone is a good prognostic marker for early-stage lung cancer, although in combination, they are highly effective at identifying patients with good prognoses. Here, we elucidate the mechanism underlying the utility of *H3F3A* and *GPR87* as prognostic markers by describing a novel process of transcriptional regulation. We evaluated the potential for these genes as prognostic markers and therapeutic targets using ectopic overexpression of *H3F3A* and an LPA antagonist, which inhibits *GPR87*. Our results indicate that using *H3F3A* as a prognostic marker and *GPR87* as a therapeutic target is a promising lung cancer treatment strategy. Considering that the combination of high *H3F3A* and *GPR87* expression is closely associated with lung cancer prognosis and that *H3F3A* epigenetically regulates *GPR87* transcription, combining LPA antagonists with epigenetic targeting of *GPR87* transcription through H3.3 could be an effective strategy for cancer treatment, including post-operative therapy for lung cancer patients to decrease lung cancer-related deaths. Therefore, we suggest that *GPR87* regulation by *H3F3A* has translational potential for novel therapeutic strategies.

## Methods

### Public data analysis

Public microarray data containing clinical information were downloaded from the NCBI GEO database (http://www.ncbi.nlm.nih.gov/geo/). The accession numbers of the collected data were GSE31210 and GSE13213. The data were globally normalized using the MAS5 method. Statistical tests were performed with the R programme, and survival analyses were performed with the R (https://www.r-project.org/) and APPEX (http://appex.kr/appex/) programs. Graphs and heatmaps were prepared using the Excel, R and MeV (http://www.tm4.org/mev.html) programs.

Public ChIP-Seq data were downloaded from the NCBI SRA (http://www.ncbi.nlm.nih.gov/sra/) and ENCODE databases (https://genome.ucsc.edu/ENCODE/). The files from the NCBI SRA database were extracted to a fastq file using the SRA toolkit programme. The accession numbers of the NCBI SRA data were SRA043915, SRP008014 (GSE31794) and SRP019254 (GSE45023). The Bowtie2 (http://bowtie-bio.sourceforge.net/bowtie2/index.shtml) and MACS (http://liulab.dfci.harvard.edu/MACS/) programs were used for mapping to the GRCh37/hg19 reference genome and peak detection. The peaks were visualized with IGV (http://www.broadinstitute.org/software/igv/) and the UCSC genome browser (http://genome.ucsc.edu/).

### Cell culture and transfection

A549 and NCI-H23 cells (ATCC) were maintained in complete RPMI 1,640 medium (Hyclone) at 37 °C in a humidified 5% CO_2_ incubator. The complete media was supplemented with 10% foetal bovine serum (Hyclone), 100 U ml^−1^ penicillin/streptomycin (WelGENE) and 2 mM L-glutamine (Hyclone).

A Flag-tagged *H3F3A* clone was constructed by PCR and inserted into the HRST-IRES-eGFP lentiviral vector. An empty vector was used as the control. The short hairpin RNA (shRNA) sequence for *H3F3A* was inserted into the pLL3.7 lentiviral vector. The target sequence of the shRNA was designed using the AsiDesigner programme (http://sysbio.kribb.re.kr:8080/AsiDesigner/). The shRNA sequences are listed in Supplementary Table 2. For viral particle generation, cloned DNAs, CMV delta 8.9 plasmid and VSV-G plasmid were transfected into 293T cells (ATCC) using Lipofectamin 2,000 (Invitrogen) in Opti-MEM media (Gibco). After 6 h of incubation, the media were changed to complete media. After 48 h of incubation, the supernatant containing the virus particles was harvested and concentrated. For viral infection, 1.5 × 10^5^ lung cancer cells were added to each well of a 6-well plate and incubated overnight. The concentrated viral supernatant was added to the plated cells, and after overnight incubation, the media were changed to complete media. After 72 h, the transfection was confirmed using a fluorescence microscope.

The siRNA target sequences were also designed using the AsiDesigner programme and the sequences of the transfected siRNAs are listed in Supplementary Table 2. For siRNA transfection, 1.5 × 10^5^ lung cancer cells were plated in each well of a 6-well plate and incubated overnight. The siRNAs or non-targeting controls at a concentration of 50 nM were transfected into lung cancer cells using Lipofectamin 2,000 in Opti-MEM media. After 4 h of incubation, the media were changed to complete media, and 48 h after transfection, the knockdown was confirmed by RT-PCR. Uncropped scans of the gel images are presented as Supplementary Fig. 12.

### Reverse transcription PCR and qPCR

Total RNA was extracted using the RNeasy Mini Kit (QIAGEN) according to the manufacturer’s instructions. Reverse transcription was performed with 1 μg of total RNA as the template and M-MLV Reverse Transcriptase (Promega). RT-PCR assays were performed using the AmfiXpand PCR Master Mix (GenDEPOT). Quantitative real-time PCR (qPCR) reactions were performed in triplicate on a LightCycler 480 machine (Roche) using the LightCycler 480 SYBR Green I Master Mix (Roche). cDNA expression was normalized to the GAPDH or β-actin levels. At least three independent biological replicates were included in each PCR/qPCR. The primers used for the PCR/qPCR reactions were designed either manually or with the Primer3 programme (http://biotools.umassmed.edu/bioapps/primer3_www.cgi). All primer sequences are listed in Supplementary Table 3. Uncropped scans of the gel images are presented as Supplementary Fig. 12.

### Immunoblot assay

Total protein samples were prepared in radioimmunoprecipitation assay buffer with protease inhibitor cocktail (Roche) and quantified using the Pierce BCA Protein Assay Kit (Thermo). Equal amounts of the samples were loaded onto 4–12% Bis-Tris Protein Gels (Novex), electrophoresed, transferred to an Immobilon-P PVDF membrane (Millipore), and probed with the antibodies listed on Supplementary Table 4. HRP-conjugated anti-mouse or anti-rabbit IgG antibody (R&D Systems) was used as the secondary antibody. The bands were detected with a WEST-ZOL plus Western Blot Detection System (iNtRON Biotechnology). Uncropped scans of the immunoblots are presented as Supplementary Fig. 12.

### Invasion assay

Transwell chambers (Corning) were coated with Matrigel Basement Membrane Matrix (BD). Cells were suspended in serum-free media and seeded into the upper chamber at a density of 2.0 to 5.0 × 10^4^ cells per well, and serum-containing media was placed into the lower chamber. After incubation for 24 h, the cells penetrating the pores were stained with Diff-Quik staining solution (Sysmex) and observed using a microscope.

### Proliferation assay

Suspensions of 1.0 to 2.0 × 10^3^ cells were seeded into 96-well. After 0–144 h of incubation at 37 °C, a mixture of CyQUANT NF Cell Proliferation dye reagent and deliverer (Invitrogen) was added. After 4 h of incubation, the ratio of the fluorescence intensity of 530 to that at 485 nm was measured.

### Microarray analysis

The Affymetrix Human ST2.0 microarray platform was used. Libraries were prepared from the total RNA samples (2 μg) and hybridized, and the intensity was measured according to the manufacturer’s instructions. The intensity values were globally normalized using the Robust Multi-array Average (RMA) method. Differential expression was tested using Student’s *t*-test and fold change. A Gene Ontology analysis was performed using DAVID (http://david.abcc.ncifcrf.gov/). Graphs and heatmaps were prepared using the Excel, R and MeV programs.

### Chromatin immunoprecipitation assays

ChIP was performed using the ChIP Assay Kit (Millipore) according to the manufacturer’s instructions. For ChIP-PCR/qPCR, 1.0 to 4.0 × 10^6^ cells were cross-linked with 1% formaldehyde for 10 min at 25 °C. For H3.3 ChIP-Seq, 3.0 × 10^7^ cells were prepared without cross-linking. The cells were resuspended and lysed for 10 min at 4 °C with the SDS Lysis Buffer provided in the kit. The lysate was aliquoted into a 1.5-ml tube and sonicated using a Bioruptor sonicator (Diagenode). The fragment size was ∼150–500 bp. Samples were diluted 10-fold (1.0 to 4.0 × 10^6^ cells per tube) in the ChIP dilution buffer provided in the kit and pre-cleared with 75 μl of Salmon Sperm DNA/Protein A Agarose beads for 30 min at 4 °C. Primary antibody was added to the pre-cleared supernatant, and the mixture was incubated overnight at 4 °C. The antibodies used for the ChIP assay are listed in Supplementary Table 4. Sixty microliters of Salmon Sperm DNA/Protein A Agarose beads were added to the samples, and the mixtures were incubated for 1 h at 4 °C. The beads were subsequently washed using wash buffers (low-salt, high-salt, LiCl and TE buffers). After 15 min of incubation at 65 °C, the precipitated chromatin was eluted twice in 250 μl of elution buffer (0.1 M NaHCO_3_ and 1% SDS). Reverse cross-linking was performed for 4 h at 65 °C. The chromatin was then treated with RNase A for 1 h at 37 °C and with Proteinase K for 1 h at 45 °C. The DNA was purified through phenol/chloroform extraction or with a QIAquick PCR Purification Kit (QIAGEN).

ChIP-PCR assays were performed using the AmfiXpand PCR Master Mix. ChIP-qPCR reactions were performed in triplicate on the LightCycler 480 machine using the LightCycler 480 SYBR Green I Master Mix. The data were normalized to the input levels. At least three independent biological replicates were included for each ChIP-PCR/qPCR. The primer sequences are listed in Supplementary Table 3. Uncropped scans of the gel images are presented as Supplementary Fig. 12.

### ChIP-Seq and RNA-Seq

Sequencing libraries for ChIP-Seq were prepared from 1 ng of DNA using the ThruPLEX-FD Prep Kit (Rubicon Genomics) according to the manufacturer’s instructions. Paired-end reads that were 76 bp in length were sequenced using a NextSeq 500 Sequencing System (Illumina). The ChIP-Seq data were analyzes using the Bowtie2 and MACS programs for mapping to the GRCh37/hg19 reference genome and peak detection. The peaks were visualized with IGV and the UCSC genome browser.

Sequencing libraries for RNA-Seq were prepared from 2 μg of total RNA using a TruSeq Stranded mRNA Sample Preparation kit (Illumina) according to the manufacturer’s instructions. Paired-end reads that were 100 bp in length were sequenced using the HiSeq 2,500 Sequencing System (Illumina). To analyse the RNA-Seq data, TopHat (http://ccb.jhu.edu/software/tophat/index.shtml) and Cufflinks (http://cufflinks.cbcb.umd.edu/manual.html) programs were used for short-read gapped alignment to the GRCh37/hg19 reference genome and *ab initio* assembly. The results were visualized with IGV and the UCSC genome browser.

### Metagene analysis of introns and promoters

Exons, introns, promoters and intergenic regions were defined based on hg19 gene annotation. The top 3,000 peaks were selected based on the peak scores provided by the MACS programme. From the top 3,000 peaks, 871 high-stringency peaks, specifically those that displayed a ChIP/input ratio that was >16 (log_2_(ChIP/input ratio)≥4), were selected. Among the 871 high-stringency peaks, intronic, exonic and promoter peaks occupied 366 gene regions. Because the generally used transcription start/end sites are not good reference positions for calculating the binding profiles of intronic peaks, we used the summit positions or peak center positions provided by the MACS programme as reference positions. To calculate the binding profiles near peaks, we divided the region near the summit positions or peak center positions (for example, −2 kb to +2 kb from H3.3 summit) into 10- or 20-bp bins and calculated the reads per kilobase per million mapped reads (RPKM) value for each bins using the Pysam Python package or HOMER (hypergeometric optimization of motif enrichment). The average RPKM of each bin was plotted using the R Plot and Lowess functions (*f*=0.3). A heatmap of the RPKM of each bin was drawn using the R and MEV programs. Boxplots for enrichment within the region were drawn using R software.

### Reporter assay

Cloned *GPR87* intronic sequences were inserted into the pGL3 Luciferase Reporter Vector (downstream of the luciferase gene with the BamHI and SalI restriction enzyme sites). A luciferase assay is performed using the Dual-Luciferase Reporter assay system (Promega) according to the manufacturer’s instructions. For transfection, 5.0 × 10^4^ cells were plated into each well of a 24-well plate. The luciferase reporters were co-transfected with the control plasmid encoding renilla luciferase into the plated cells. The luciferase activity was measured using a VICTOR Light luminometer (PerkinElmer). The transfection efficiencies were normalized using the renilla luciferase activity as the control.

### Chromosome conformation capture (3C)

First, 5.0 × 10^4^ cells were cross-linked with 2% formaldehyde for 10 min at 25 °C. Five millilitres of NP-40 buffer (10 mM Tris-HCl, pH 7.5, 10 mM NaCl, 0.2% NP-40, protease inhibitor cocktail) were added to the cells, and the cells were incubated at 4 °C for 2 h. The mixture was then centrifuged, and the pellet was resuspended in 0.5 ml of 1.2x DpnII restriction enzyme buffer (New England Biolabs). Fifteen microliters of 10% SDS was added to the sample, and the mixture was incubated at 37 °C for 1 h. Forty microliters of 25% Triton-X100 was added, and the sample incubated at 37 °C for 1 h. The sample was digested with 400 units of DpnII restriction enzyme at 37 °C for ∼18 h. To deactivate DpnII, 80 μl of 10% SDS was added to the sample, and the mixture was incubated at 65 °C for 20 min. Then, 6.125 ml of 1.15 × ligation buffer and 300 μl of 25% Triton-X100 were added, and the sample was incubated at 37 °C for 1 h. The digested samples were ligated with 100 units of T4 DNA ligase (Promega) at 16 °C for 4 h and then at 37 °C for 30 min. Reverse cross-linking was performed for overnight at 65 °C. The chromatin was then treated with RNase A for 1 h at 37 °C and with Proteinase K for 1 h at 45 °C. The DNA was purified through phenol/chloroform extraction or with the QIAquick PCR Purification Kit.

3C-PCR assays were performed using the AmfiXpand PCR Master Mix. The data were normalized to ‘internal’ primers located in the GAPDH gene. At least three independent biological replicates were included for each 3C-PCR assay. The primer sequences are listed in Supplementary Table 3. Uncropped scans of the gel images are presented as Supplementary Fig. 12.

### Data availabilty

Sequencing data have been deposited in the SRA with the accession number SRP078775. Micoarray data have been deposited in the GEO with the accession number GSE84555. All the relevant data are available from the corresponding author (Y.-J.K.).

## Additional information

**How to cite this article:** Park, S.-M. *et al*. Histone variant *H3F3A* promotes lung cancer cell migration through intronic regulation. *Nat. Commun.* **7,** 12914 doi: 10.1038/ncomms12914 (2016).

## Supplementary Material

Supplementary Information Supplementary Figures 1-12 and Supplementary Tables 1-4.

Supplementary Data 1 DEGs which show positively correlated expression pattern with *H3F3A*

Supplementary Data 2 DEGs which show negatively correlated expression pattern with *H3F3A*

Supplementary Data 3 GO analysis result of DEGs which show positively correlated expression pattern with *H3F3A*

Supplementary Data 4 GO analysis result of DEGs which show negatively correlated expression pattern with *H3F3A*

## Figures and Tables

**Table 1:** Multivariate Cox proportional hazard analysis for the prediction of stage I lung adenocarcinoma patient survival.

**Variable**	**Survival**	
	**HR (95% CI)**	***P*** **value**
*GSE13213 overall*		
*H3F3A*	2.80 (1.11–7.03)	**0.0288**
*p53* mutation	1.71 (0.251–1.36)	0.212
Stage IA versus IB	1.68 (0.718–3.94)	0.231
*EGFR* mutation	1.63 (0.604–4.40)	0.334
Smoking	1.00 (0.999–1.00)	0.504
*H3F3B*	1.25 (0.340–1.89)	0.614
*K-ras* mutation	1.29 (0.228–2.66)	0.689
		
*GSE31210 overall*		
*H3F3A*	5.71 (1.24–26.3)	**0.0256**
*H3F3B*	3.80 (1.04–13.9)	**0.0434**
EGFR mutation	2.03 (0.174–1.39)	0.182
Stage IA versus IB	1.52 (0.53856–4.267)	0.431
MYC expression	1.46 (0.294–7.26)	0.644
MYC copy	1.24 (0.399–3.84)	0.711
K-ras mutation	1.49 (0.0783–5.77)	0.717
Smoking	1.18 (0.287–2.50)	0.765
ALK fusion	1.47 × 10-7 (0–∞)	1.00
		
*GSE31210 relapse-free*		
*H3F3A*	6.68 (1.46–30.4)	**0.0141**
*H3F3B*	3.24 (0.885–11.9)	0.0758
EGFR mutation	2.10 (0.169–1.34)	0.159
Stage IA versus IB	1.90 (0.653–5.51)	0.240
K-ras mutation	1.52 (0.0774–5.60)	0.702
MYC copy	1.06 (0.337–3.36)	0.916
Smoking	1.05 (0.322–2.82)	0.930
MYC expression	1.07 (0.183–4.77)	0.935
ALK fusion	4.91 × 10-7 (0–∞)	1.00

CI, confidence interval; HR, hazard ratio.

Bold entries indicate *P*<0.05.
