# Supplementary material for: Histone variant H3F3A promotes lung cancer cell migration through intronic regulation
Source: Nat Commun. 2016 Oct 3;7:12914. doi: 10.1038/ncomms12914 (PMC5477500; doi:10.1038/ncomms12914)
Supplement: Supplementary Information — Supplementary Figures 1-12 and Supplementary Tables 1-4. [file ncomms12914-s1.pdf]

Supplementary Figures

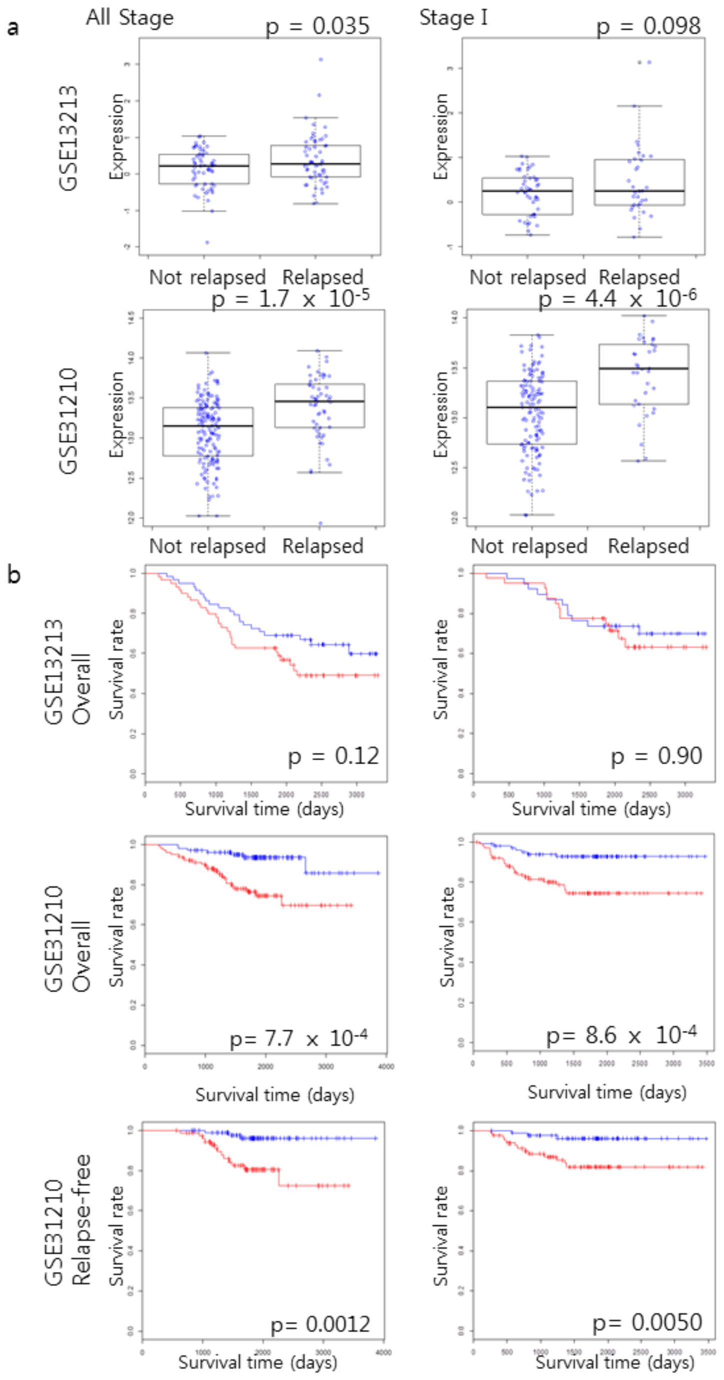

Supplementary Figure 1. H3F3B expression in lung cancer. a. Comparison of *H3F3B* expression in relapsed and non-relapsed lung cancer patients. b. Prognosis of two groups of lung cancer patients classified by *H3F3B* expression. (Red: high expression group, Blue: low expression group).

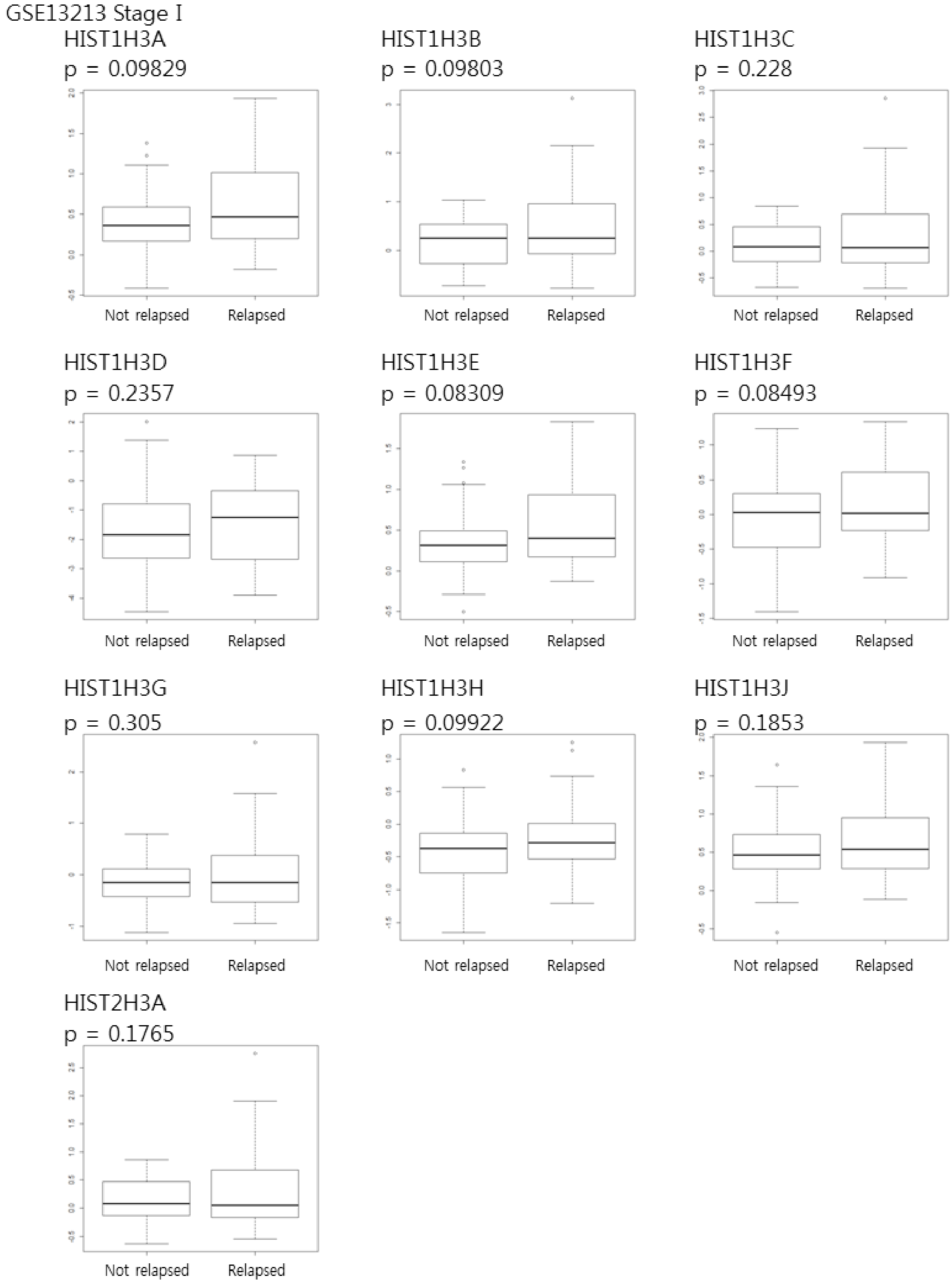

Supplementary Figure 2. Comparison of canonical histone expression in relapsed and non-relapsed lung cancer patients

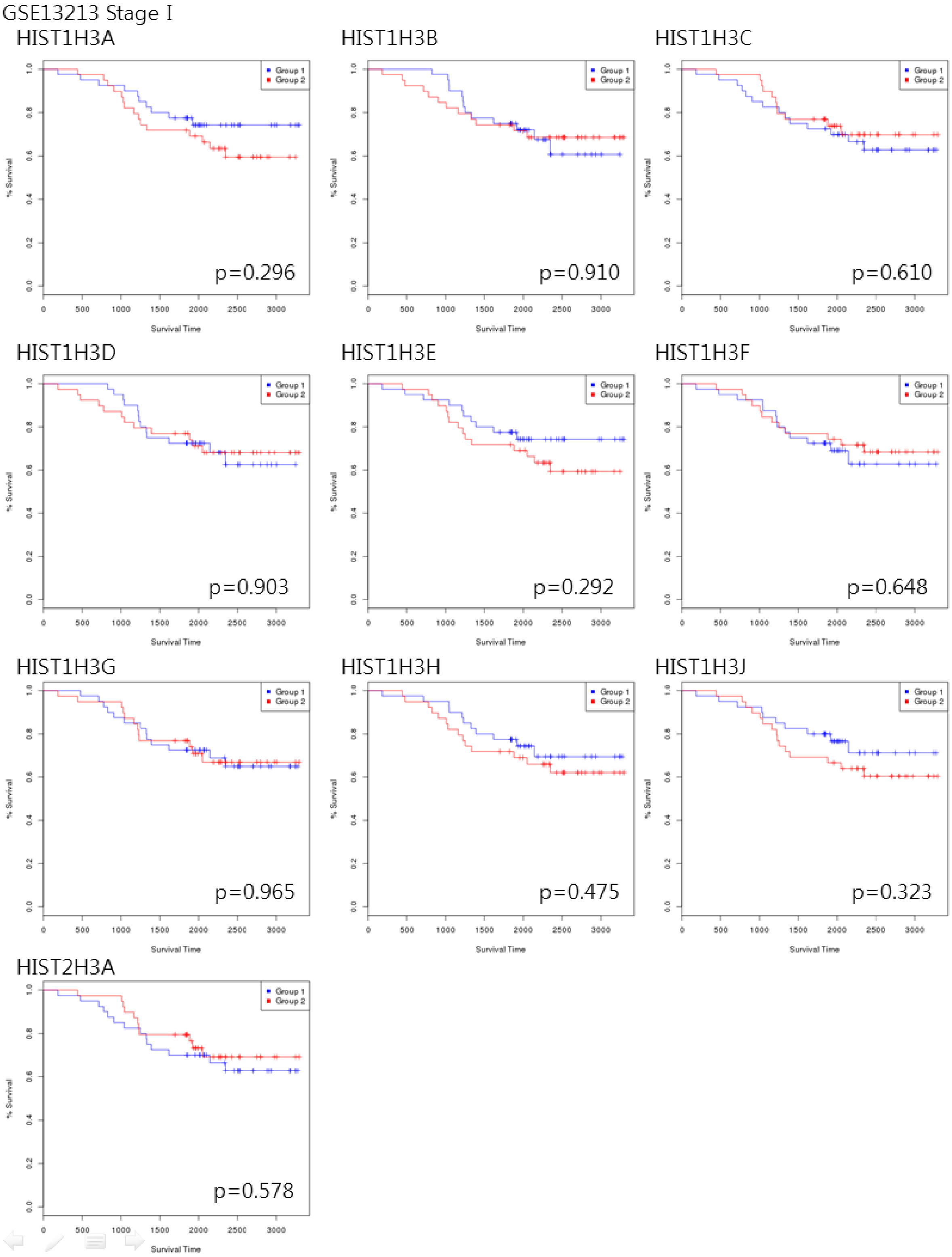

Supplementary Figure 3. Prognosis of two groups of lung cancer patients classified by canonical histone expression. (Red: high expression group, Blue: low expression group)

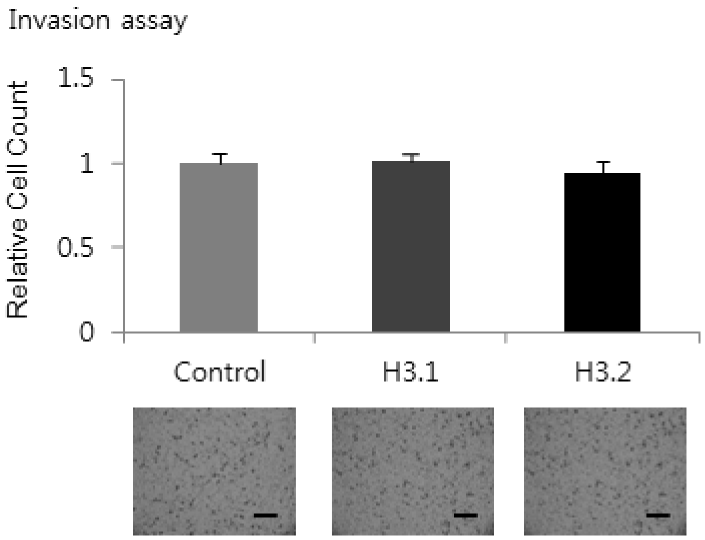

Supplementary Figure 4. The results of invasion assays using canonical histone H3-overexpressing A549 cells. Scale bar, 200μm.

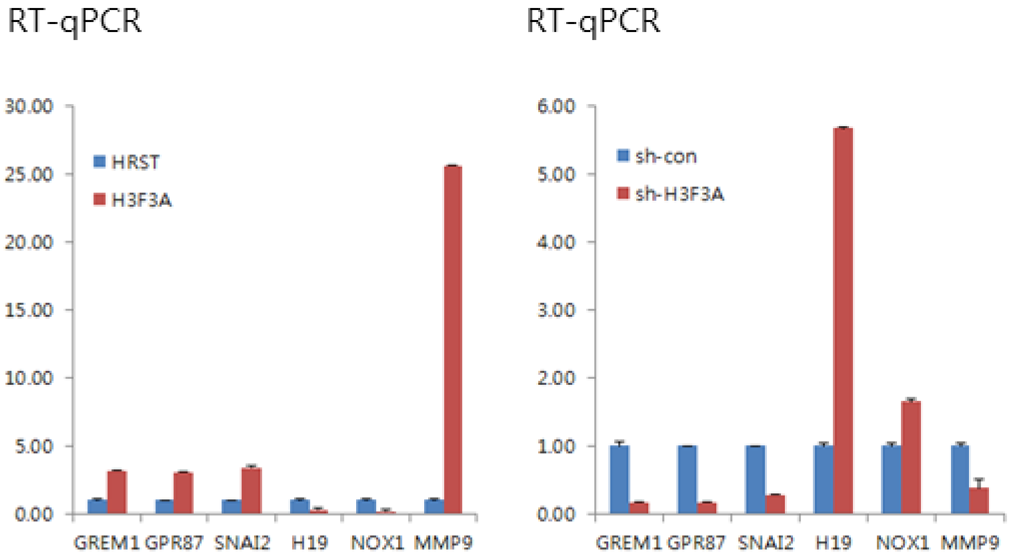

Supplementary Figure 5. Validation of DEGs using RT-qPCR.

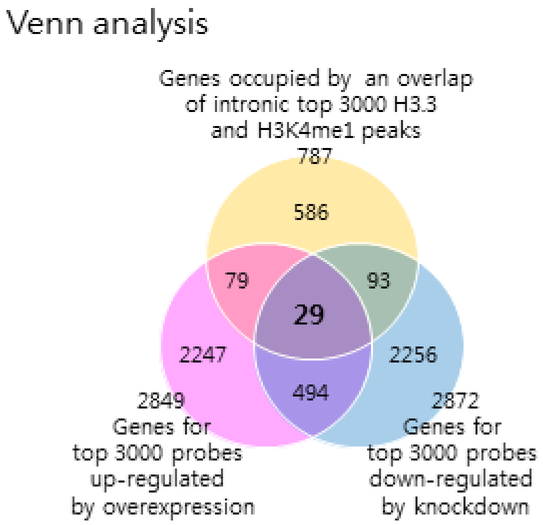

Supplementary Figure 6. Venn analysis using H3.3 ChIP-seq and microarray data.

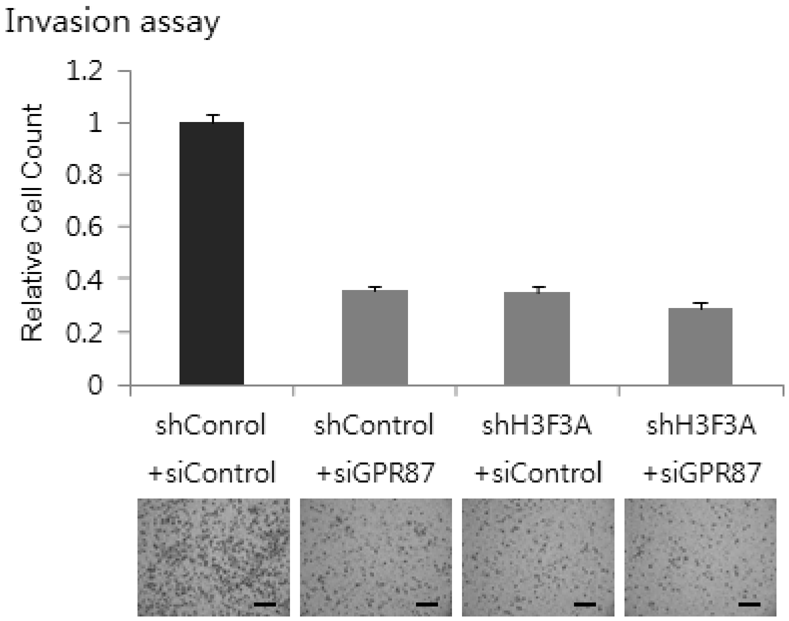

Supplementary Figure 7. The results of invasion assays after H3.3/GPR87 single and double knockdown. Scale bar, 200μm.

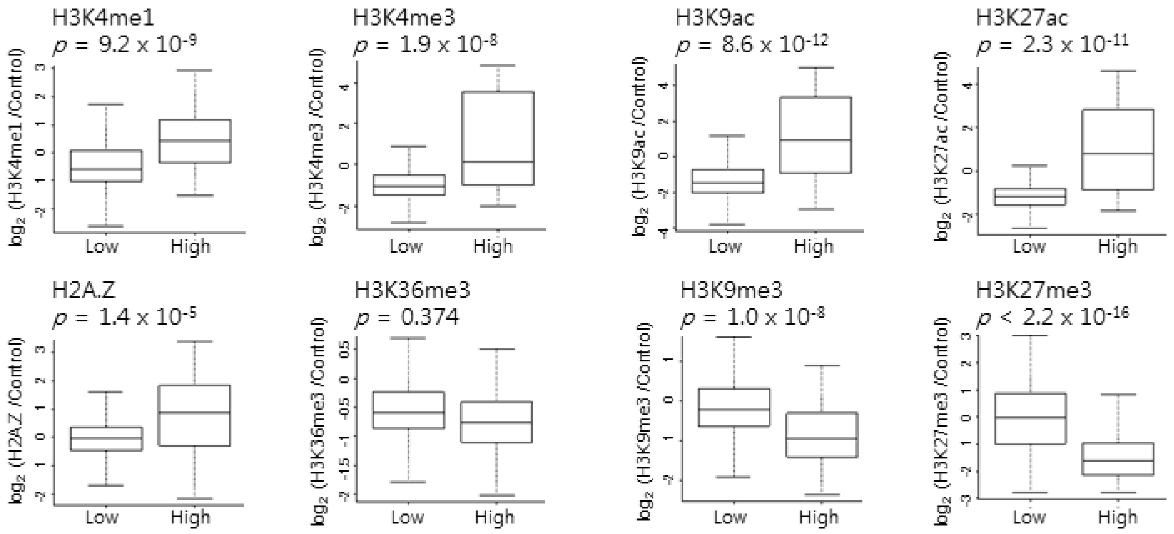

Supplementary Figure 8. Comparison of histone marks on H3.3 peaks between low turnover and high turnover peaks defined by SRA043915 dataset (Huang et al. 2013).

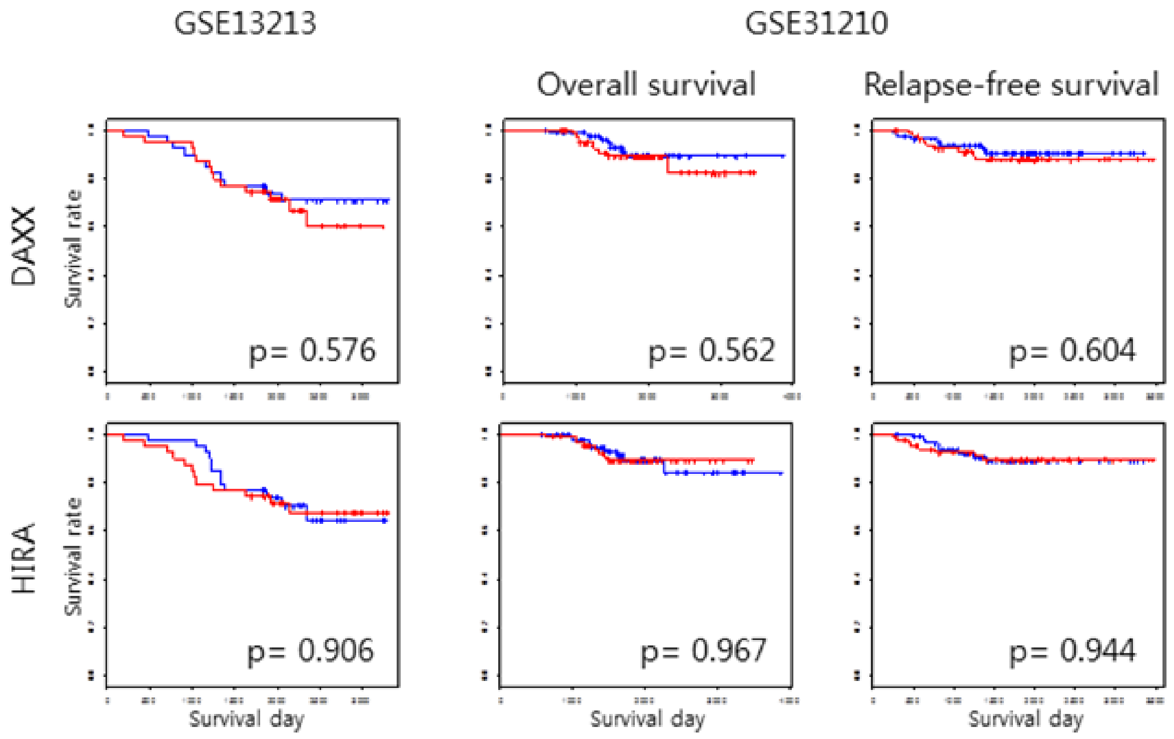

Supplementary Figure 9. Prognosis of two groups of lung cancer patients classified by *DAXX* and *HIRA* expression. (Red: high expression group, Blue: low expresssion group).

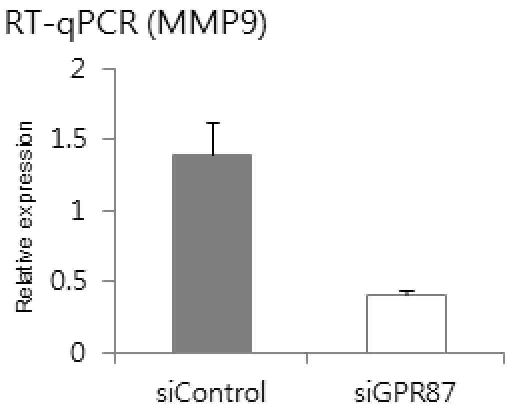

Supplementary Figure 10. Measuring MMP9 expression after GPR87 knockdown using RT-qPCR assay. Scale bar, 200μm.

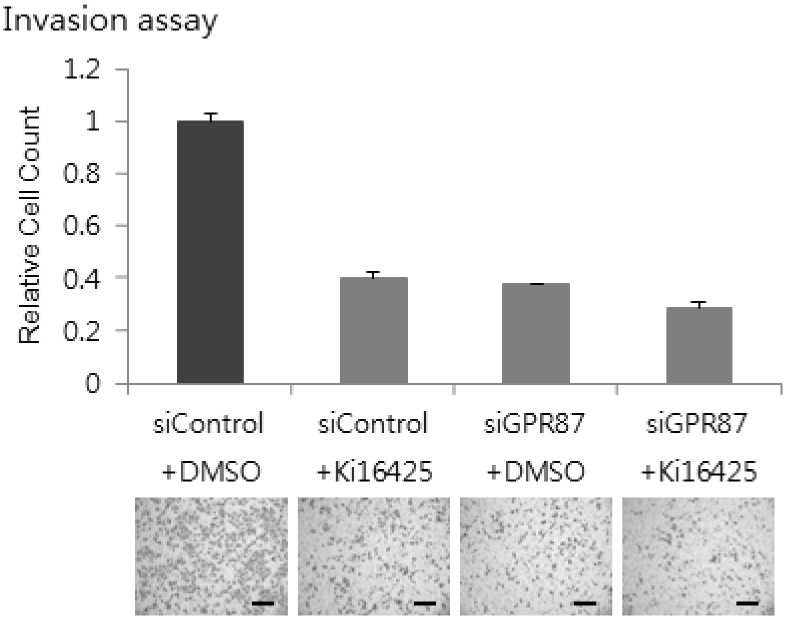

Supplementary Figure 11. The results of invasion assays after GPR87 knockdown and/or Ki16425 treatment. Scale bar, 200μm.

Figure 1a and c Immunoblot

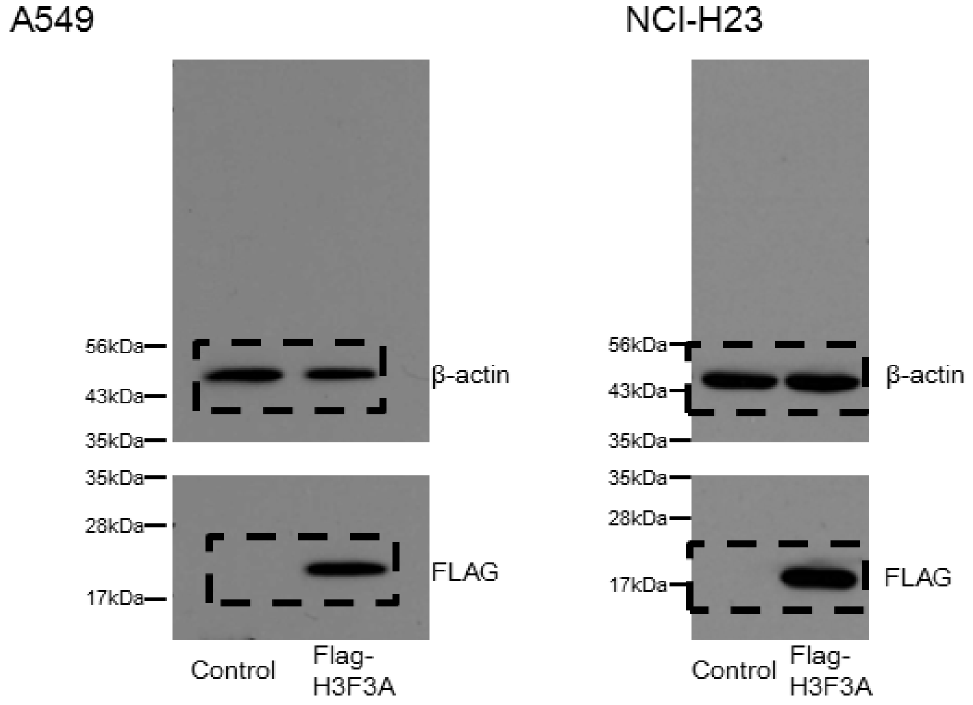

Figure 1b and d RT-PCR

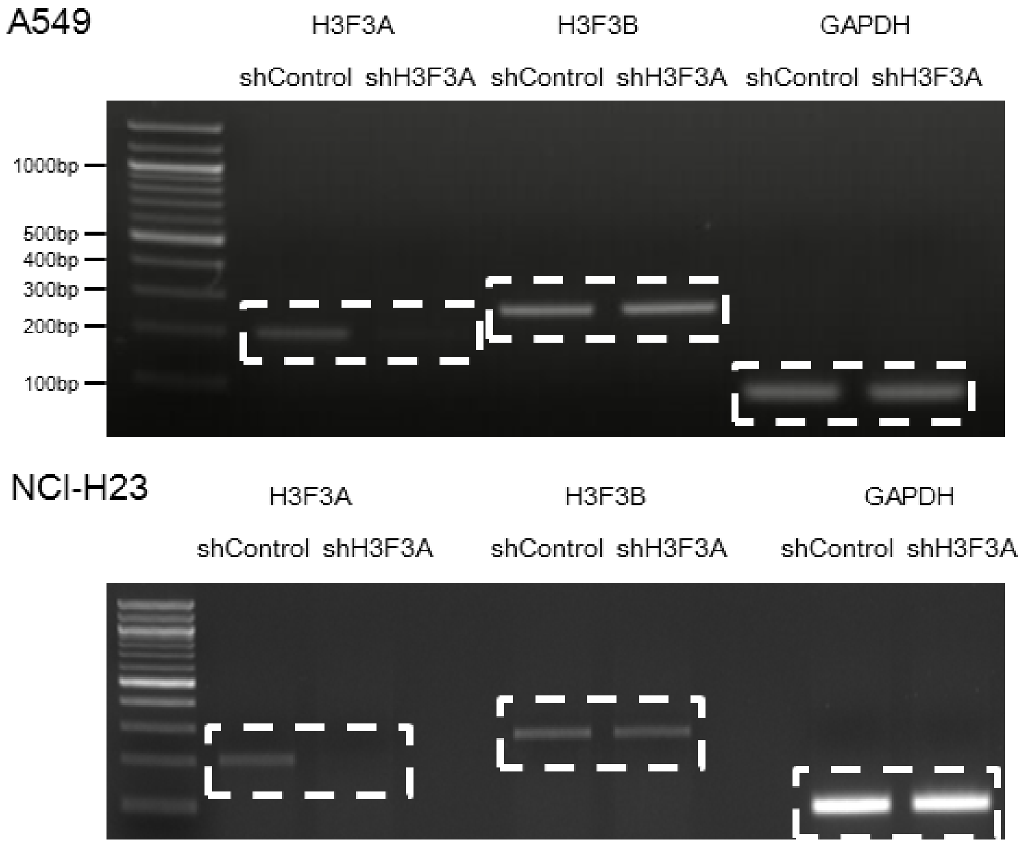

Figure 3c RT-PCR

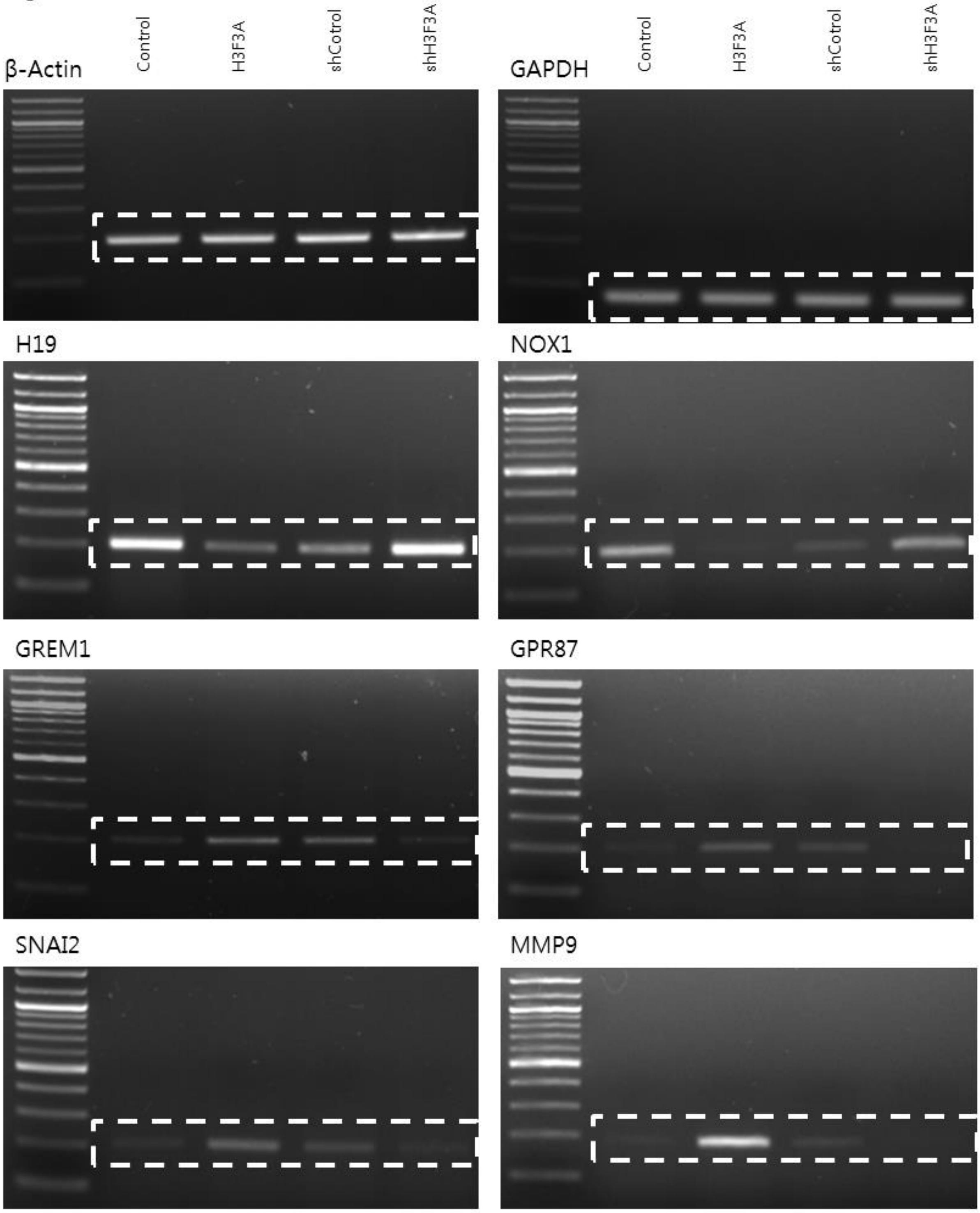

Figure 5c ChIP-PCR

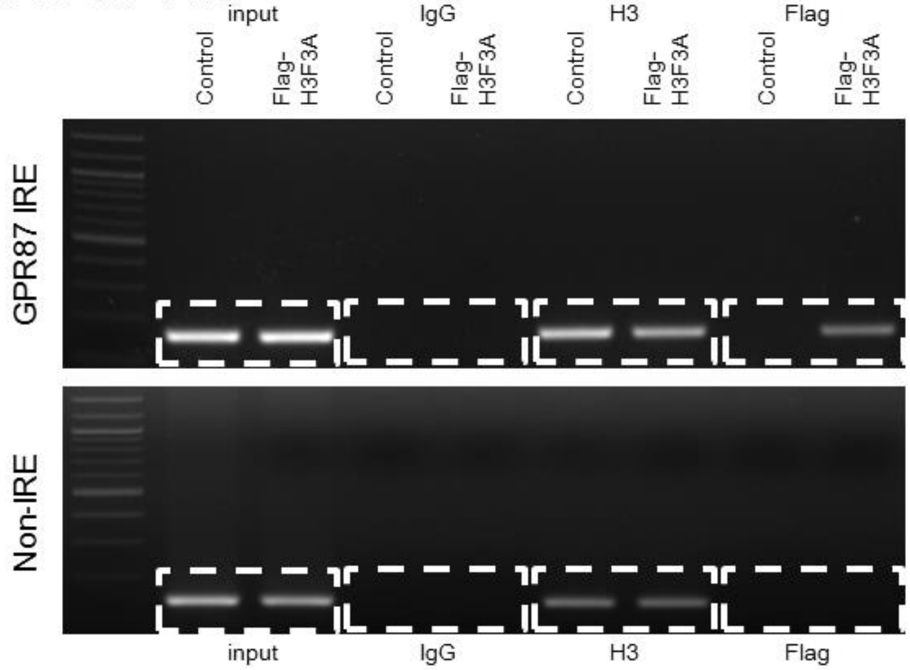

Figure 6e 3C PCR

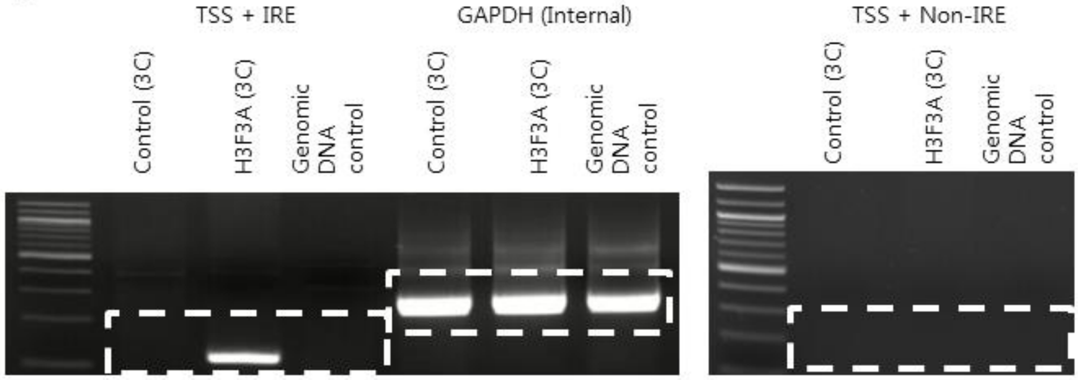

Figure 7C RT-PCR

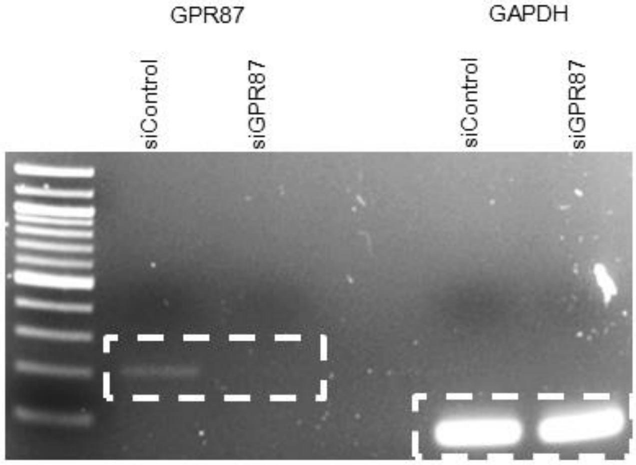

Supplementary Figure 12. Uncropped scans of the blots and gel images

Supplementary Tables

Supplementary Table 1. Result of log-rank test between the patients in each group and out of the group. The groups were divided by *H3F3A* and *GPR87* expression level.

Group	p by log-rank test (In group vs. out of group)	
	GSE13213	GSE31210
H3F3A_low, GPR87_low	**0.002030**	**0.016900**
H3F3A_low, GPR87_high	0.332000	0.152000
H3F3A_high, GPR87_low	0.938000	0.923000
H3F3A_high, GPR87_high	**0.013900**	**0.000009**

Supplementary Table 2. Sequences of shRNAs and siRNAs used in this study

Name	sequence
shControl-F	TGC CTA CGC CAC CAA TTT CGT TTC AAG AGA ACG AAA TTG GTG GCG TAG GTT TTT TC
shControl-R	TCG AGA AAA AAC CTA CGC CAC CAA TTT CGT TTC AAG AGA ACG AAA TTG GTG GCG TAG GCA
shH3F3A-F	TGT GAA GAA ACC TCA TCG TTA TTC AAG AGA TAA CGA TGA GGT TTC TTC ATT TTT TC
shH3F3A-R	TCG AGA AAA AAT GAA GAA ACC TCA TCG TTA TTC AAG AGA TAA CGA TGA GGT TTC TTC ACA
siControl-F	CCU ACG CCA CCA AUU UCG U(dTdT)
siControl-R	ACG AAA UUG GUG GCG UAG G(dTdT)
siGPR87-F	GAC CUU AGU UUC AAA GCU U(dTdT)
siGPR87-R	AAG CUU UGA AAC UAA GGU C(dTdT)
siHIRA-F	CUG ACA AGC AGG ACU CAC U(dTdT)
siHIRA-R	AGU GAG UCC UGC UUG UCA G(dTdT)
siDAXX-F	CUG AUA CCU UCC CUG ACU A(dTdT)
siDAXX-R	UAG UCA GGG AAG GUA UCA G(dTdT)

Supplementary Table 3. Sequences of primers used in this study

Name	sequence
**RT-PCR and RT-qPCR**	
H3F3A_F	GGC TCG TAC AAA GCA GAC TGC C
H3F3A_R	GGA AGT TTG CGA ATC AGA AG
H3F3B_F	CCA AGC AGA CTG CTC GTA AGT C
H3F3B_R	CGA TGG CTG CGC TCT GAA ACC
GPR87_F	GGG GAG ATG TTT CGT TTT CA
GPR87_R	AAC ACT TGG GGA CGA TTG AG
MMP9_F	CTC GAA CTT TGA CAG CGA CA
MMP9_R	GCC ATT CAC GTC GTC CTT AT
β-Actin_F	TCC CTG GAG AAG AGC TAC GA
β-Actin_R	AGC ACT GTG TTG GCG TAC AG
GAPDH_F	TCC TGC ACC ACC AAC TGC TTA G
GAPDH_R	TGG TCA TGA GTC CTT CCA CGA TAC
**ChIP PCR and ChIP qPCR**	
GPR87_IRE_F	CCT GGA ACC AAG CCC TTT AC
GPR87_IRE_R	ACC TGC CTG CTG TCT TTC TG
Non_IRE_F	CCA CAA AAA TGC AGG AAC TAC A
Non_IRE_R	TCC TCC ACT CTC CAG TTT TCC
GPR87 proximal promoter (-113 ~ +36 bp from TSS) F	GCG ACT ACT GGA ACA ACT TGC
GPR87 proximal promoter (-113 ~ +36 bp from TSS) R	CTA AGC TCC AGC CCT CTG TG
GPR87 distal promoter (-898 ~ -733 bp from TSS) F	GGT AGG ATT ACA GGT GCC CAC GAC T
GPR87 distal promoter (-898 ~ -733 bp from TSS) R	CGG GCG CAG TGG CTC ACA CA
**3C PCR**	
TSS	CGT GGG CAC CTG TAA TCC TAC
IRE	GCC ATC TCA GCA TAA ACA AGG
Non-IRE	TCA GAT GGC TTT TGT GAA GAC
GAPDH (internal) F	ACA GTC CAT GCC ATC ACT GCC
GAPDH (internal) R	GCC TGC TTC ACC ACC TTC TTG

Supplementary Table 4. Antibodies used in this study

Antigen	Company	Catalog #	Description
Flag	Sigma	F1804	Monoclonal ANTI-FLAG M2 antibody
β-actin	Cell Signaling	5125s	β-Actin (13E5) Rabbit mAb (HRP Conjugate)
IgG	Cell Signaling	12-370	Polyclonal Rabbit anti-IgG antibody
H3	Abcam	ab1791	Anti-Histone H3 antibody
H2A.Z	Millipore	07-594	Anti-Histone H2A.Z Antibody
H3K4me1	Abcam	ab8895	Anti-Histone H3 (mono methyl K4) antibody
H3K9ac	Abcam	ab4441	Anti-Histone H3 (acetyl K9) antibody
Pol II	BioLegend	920101	Anti-RNA Polymerase II
Pol II phospho S5	Abcam	ab5408	Anti-RNA polymerase II CTD repeat YSPTSPS (phospho S5) antibody [4H8]
